# Supplementary material for: Expanding the limits of nuclear stability at finite temperature
Source: Nat Commun. 2023 Aug 10;14:4834. doi: 10.1038/s41467-023-40613-2 (PMC10415286; doi:10.1038/s41467-023-40613-2)
Supplement: Supplementary file 1 — Supplementary Information [file 41467_2023_40613_MOESM1_ESM.pdf]

# Supplementary Information

# Expanding the limits of nuclear stability at finite temperature

Ante Ravlić[1],* Esra Yüksel[2], Tamara Nikšić[1], and Nils Paar[1]†

[1]*Department of Physics, Faculty of Science, University of Zagreb, Bijenička c. 32, 10000 Zagreb, Croatia and*

[2]*Department of Physics, University of Surrey, Guildford, Surrey, GU2 7XH, United Kingdom*

* aravlic@phy.hr

† npaar@phy.hr

## Supplementary Note 1. THEORETICAL FORMALISM

We consider the nucleus at finite temperature within the relativistic energy density functional (EDF) formalism. The most common approach at finite temperatures is to solve the finite-temperature relativistic mean-field equations (FT-RMF). However, with non-vanishing temperatures, a proper treatment of the continuum states is mandatory to achieve convergence of our results. Therefore, we implement the method first introduced by Bonche, Levit and Vautherin in Refs. [1, 2], further denoted as the BLV prescription. Finally, we generalize the BLV prescription to treat superfluid nuclei within the finite-temperature relativistic Hartree-Bogoliubov (FT-RHB) theory, including also the deformation and pairing in the calculations.

### A. The FT-RMF theory

The FT-RMF equations are derived by assuming the nucleus is an isolated system that cannot exchange heat or particles with the environment [3]. Such a system is characterized by its temperature $T$ and chemical potential $\lambda$. In order to derive the FT-RMF equations, we start by defining the grand-canonical potential $\Omega$

$$\Omega = E - TS - \sum_q \lambda_q N_q, \tag{S1}$$

where the internal energy is $E = \mathrm{Tr}\left[\hat{D}\hat{H}\right]$, entropy is $S = -k_\mathrm{B}\mathrm{Tr}\left[\hat{D}\ln\hat{D}\right]$ and particle number is $N = \mathrm{Tr}\left[\hat{D}\hat{N}\right]$, $\hat{D}$ being the single-particle density operator and $k_\mathrm{B}$ is the Boltzmann constant. The chemical potential is defined both for protons ($\lambda_\mathrm{p}$) and neutrons ($\lambda_\mathrm{n}$). The many-body Hamiltonian operator assumes the usual form [4]

$$\hat{H} = \sum_{ij} t_{ij} c_i^\dagger c_j + \frac{1}{4}\sum_{ijkl} \bar{v}_{ijkl} c_i^\dagger c_j^\dagger c_l c_k, \tag{S2}$$

where $t_{ij}$ is the kinetic energy and $\bar{v}_{ijkl} = v_{ijkl} - v_{ijlk}$ anti-symmetrized two-body interaction, while $c_k^\dagger, c_k$ are the single-particle creation and annihilation operators, respectively. The gist of the FT-RMF theory is to assume an ensemble of independent-particles; thus, the density operator is approximated by the FT-RMF density operator [3]

$$\hat{D}_\mathrm{RMF} = \prod_i \left[f_i \hat{n}_i + (1-f_i)(1-\hat{n}_i)\right], \tag{S3}$$

where the Fermi-Dirac factor is defined as $f_i = [1 + \exp[\beta(\varepsilon_i - \lambda_q)]]^{-1}$, $\beta = 1/(k_{\mathrm{B}}T)$, while $\varepsilon_i$ is the single-particle energy. The number operator of a particular state is defined as $\hat{n}_i = c_i^\dagger c_i$. Now, it is straightforward to show that

$$\rho_{ij} = \mathrm{Tr}\left[\hat{D}_{\mathrm{RMF}} c_j^\dagger c_i\right] = f_i \delta_{ij}, \tag{S4}$$

which is also known as the generalized Wick contraction [5]. By evaluating the respective traces within the FT-RMF approximation, we can rewrite the grand-canonical potential as

$$\Omega = \mathrm{Tr}\left[\left(t + \frac{1}{2}\Gamma - \lambda_q\right)\rho\right] + k_{\mathrm{B}}T\sum_k \left[(1-f_k)\mathrm{ln}(1-f_k) + f_k \mathrm{ln} f_k\right], \tag{S5}$$

with the usual notation $\Gamma_{ij} = \sum_{kl} \bar{v}_{ikjl}\rho_{lk}$. We perform variations over the density $\rho$ and Fermi-Dirac factors $f$ to get [3]

$$\delta\Omega = \mathrm{Tr}\left[(t - \lambda_q)\delta\rho\right] + \mathrm{Tr}\left[\Gamma\delta\rho\right] + k_{\mathrm{B}}T\sum_k (\delta f_k)\mathrm{ln}\frac{f_k}{1-f_k}. \tag{S6}$$

By defining $\varepsilon_i = -k_{\mathrm{B}}T\sum_k (\delta f_k)\mathrm{ln}\frac{f_k}{1-f_k}$, we obtain the FT-RMF equations in the well-known form

$$\delta\Omega = \mathrm{Tr}\left[(h - \varepsilon)\delta\rho\right] = 0, \tag{S7}$$

with the mean-field Hamiltonian defined as $h = t + \Gamma - \lambda_q$. Within the RMF formalism above equation takes a form of the single-particle Dirac equation [6, 7]

$$\left[-i\boldsymbol{\alpha}\boldsymbol{\nabla} + \beta M^*(\boldsymbol{r}) + V(\boldsymbol{r})\right]\psi_i(\boldsymbol{r}) = \varepsilon_i \psi_i(\boldsymbol{r}), \tag{S8}$$

where the effective mass is defined as $M^*(\boldsymbol{r}) = m + S(\boldsymbol{r})$, $S(\boldsymbol{r})$ is the scalar potential, $V(\boldsymbol{r})$ the vector potential and $m$ the bare nucleon mass. The single-particle wavefunction $\psi_i(\boldsymbol{r})$ has the form of a two component Dirac spinor $\begin{pmatrix} f_i(\boldsymbol{r}) & ig_i(\boldsymbol{r}) \end{pmatrix}^T$, with $f_i(g_i)$ being the upper(lower) component. In this work, we assume two main methods to solve Eq. (S8):

- discretization in the basis of the harmonic oscillator. The Dirac spinor can be expanded as

$$f_i(\boldsymbol{r}, s) = \sum_{n=0}^{n_{\max}} f_n^{(i)}\Phi_n(\boldsymbol{r}, s), \quad g_i(\boldsymbol{r}, s) = \sum_{\tilde{n}=0}^{\tilde{n}_{\max}} g_{\tilde{n}}^{(i)}\Phi_{\tilde{n}}(\boldsymbol{r}, s), \tag{S9}$$

  where $s$ denotes the spin coordinate and $\Phi_n(\boldsymbol{r}, s)$ is the harmonic oscillator wavefunction. The expansion in upper(lower) components is cut-off at some shell number $n_{\max}(\tilde{n}_{\max})$.

- finite-element method based on the expansion of wavefunctions in a proper basis of interpolating polynomials in the coordinate-space:

$$f_i(\boldsymbol{r},s) = \sum_n f_n^{(i)} B_n^{(j)}(\boldsymbol{r},s), \quad g_i(\boldsymbol{r},s) = \sum_{\tilde{n}} g_{\tilde{n}}^{(i)} B_{\tilde{n}}^{(j)}(\boldsymbol{r},s), \tag{S10}$$

  where $B_n^{(j)}(\boldsymbol{r},s)$ is a generalized interpolation function of $j$-th order.

For now, we keep the equations general and later we will assume a specific symmetry of a nucleus: either spherical or axially-deformed. Details of the vector $V$ and scalar $S$ fields depend on the selected functional. In this work, it is either the meson-exchange (DD-ME2) or the point-coupling (DD-PC family).

### B. Continuum subtraction — the BLV prescription

As the temperature increases, a non-vanishing number of single-particle states gets scattered to the particle continuum. In our work, we employ the prescription developed in Refs. [1, 2], which we denote as the BLV prescription in the following. Within the BLV prescription, we can define two density matrices: (i) $\rho$ density matrix, associated with the system consisting of a nucleus and a surrounding vapor (Nucl+Vap), and (ii) $\tilde{\rho}$ which corresponds to the vapor solution (Vap) only. Minimization is performed with the subtracted grand-canonical potential defined as

$$\Delta\Omega = \Omega(\rho) - \Omega(\tilde{\rho}). \tag{S11}$$

By inserting Eq. (S5), we obtain:

$$\Delta\Omega = \mathrm{Tr}\left[(t-\lambda)\bar{\rho}\right] + \frac{1}{2}\mathrm{Tr}\left[\Gamma\rho - \tilde{\Gamma}\tilde{\rho}\right] + T\bar{S}, \tag{S12}$$

where we define the subtracted density as $\bar{\rho} = \rho - \tilde{\rho}$. The subtracted entropy is given by

$$\begin{aligned} \bar{S} = &-k_{\mathrm{B}} \sum_k \left[(1-f_k)\ln(1-f_k) + f_k \ln f_k\right] \\ &+ k_{\mathrm{B}} \int d\varepsilon g(\varepsilon) \left[(1-f(\varepsilon))\ln(1-f(\varepsilon)) + f(\varepsilon)\ln f(\varepsilon)\right]. \end{aligned} \tag{S13}$$

The summation is performed over the discrete single-particle states (denoted by $k$) and integration is over the continuum states with level density $g(\varepsilon)$. However, in practical calculations, continuum is often discretized, therefore we replace the integration with summation

over the continuum states. Using the short-hand notation of Ref. [4], we rewrite the second term in Eq. (S12) as

$$\frac{1}{2}\mathrm{Tr}\left[\Gamma\rho - \tilde{\Gamma}\tilde{\rho}\right] = \frac{1}{2}\mathrm{Tr}_1\mathrm{Tr}_1(\bar{\rho}\bar{v}\bar{\rho}) + \mathrm{Tr}_1\mathrm{Tr}_1(\bar{\rho}\bar{v}\tilde{\rho}). \tag{S14}$$

The chemical potential $\lambda_q$ is fixed in order to reproduce the total particle number $N_q$

$$\int d\boldsymbol{r}\bar{\rho}(\boldsymbol{r}) = N_q. \tag{S15}$$

First, to get the FT-RMF equation for the Nucl+Vap system, we vary Eq. (S12) with respect to the Nucl+Vap density $\rho$

$$\delta_\rho \Delta\Omega = \mathrm{Tr}\left[(t-\lambda)\delta\rho\right] + \mathrm{Tr}_1\mathrm{Tr}_1(\rho\bar{v}\delta\rho) + T\frac{\delta\bar{S}}{\delta\rho}\delta\rho, \tag{S16}$$

and the FT-RMF equation for the Vap system is obtained by variation with respect to $\tilde{\rho}$

$$\delta_{\tilde{\rho}} \Delta\Omega = \mathrm{Tr}\left[(t-\lambda)\delta\tilde{\rho}\right] + \mathrm{Tr}_1\mathrm{Tr}_1(\tilde{\rho}\bar{v}\delta\tilde{\rho}) + T\frac{\delta\bar{S}}{\delta\tilde{\rho}}\delta\tilde{\rho}. \tag{S17}$$

The coupling between the densities of these two systems is given through Eq. (S15). However, in Refs. [1, 2] it is noted that the Coulomb term has to be treated separately in order to avoid divergences, stemming from the long-range charged vapor contribution. The BLV prescription imposes the following form of the Coulomb interaction term

$$\begin{aligned}&\frac{1}{2}\mathrm{Tr}_1\mathrm{Tr}_1(\bar{\rho}\bar{v}\bar{\rho}) + \mathrm{Tr}_1\mathrm{Tr}_1(\bar{\rho}\bar{v}\tilde{\rho})\\ &\to \frac{1}{2}\mathrm{Tr}_1\mathrm{Tr}_1(\rho\bar{v}\rho) - \frac{1}{2}\mathrm{Tr}_1\mathrm{Tr}_1(\tilde{\rho}\bar{v}\tilde{\rho}) - \mathrm{Tr}_1\mathrm{Tr}_1(\tilde{\rho}\bar{v}\bar{\rho}).\end{aligned} \tag{S18}$$

Components of the nuclear interaction remain unchanged. For demonstration purposes, in the following, we assume that the only interaction term at the mean-field level is the direct Coulomb term (and denote it by $v^c$ with the corresponding proton density $\rho_{\mathrm{p}}$). Varying Eq. (S12) with $\rho$ and $\tilde{\rho}$ gives us the FT-RMF equations for the Nucl+Vap and Vap system, respectively

$$\begin{aligned}\delta_\rho \Delta\Omega &= \mathrm{Tr}\left[(t-\lambda)\delta\rho\right] + \mathrm{Tr}_1\mathrm{Tr}_1(\bar{\rho}_p v^c\delta\rho) + T\frac{\delta\bar{S}}{\delta\rho}\delta\rho,\\ \delta_{\tilde{\rho}} \Delta\Omega &= \mathrm{Tr}\left[(t-\lambda)\delta\tilde{\rho}\right] + \mathrm{Tr}_1\mathrm{Tr}_1(\bar{\rho}_p v^c\delta\tilde{\rho}) + T\frac{\delta\bar{S}}{\delta\tilde{\rho}}\tilde{\rho}.\end{aligned} \tag{S19}$$

Therefore, within the BLV subtraction procedure, the self-consistent FT-RMF problem consists of two systems: (i) Nucl+Vap and (ii) Vap only, which are coupled by the direct Coulomb term $\sum_{kl} v^c_{ijkl}\bar{\rho}_{p_{kl}}$.

### C. Introduction of pairing correlations at finite temperature

The pairing interaction can be included in a straightforward manner by introducing the Bogoliubov transformation of single-particle operators [4]

$$c_k = \sum_l U_{kl}\beta_l + V^*_{kl}\beta^\dagger_l, \quad c^\dagger_k = \sum_l V_{kl}\beta_l + U^*_{kl}\beta^\dagger_l, \tag{S20}$$

where $\beta^\dagger_k, \beta_k$ are the quasi-particle (q.p.) creation and annihilation operators, respectively. We can now introduce the pairing tensor $\kappa$, which together with the single-particle density in the single-particle basis $\{c^\dagger_k, c_k\}$ assumes the form [3]

$$\begin{aligned} \rho &= UfU^\dagger + V^*(1-f)V^T, \\ \kappa &= UfV^\dagger + V^*(1-f)U^T. \end{aligned} \tag{S21}$$

By introducing the pairing correlations, Eq. (S12) can be rewritten as

$$\Delta\Omega = \mathrm{Tr}\left[(t-\lambda)\bar{\rho}\right] + \frac{1}{2}\mathrm{Tr}\left[\Gamma\rho - \tilde{\Gamma}\tilde{\rho}\right] + \frac{1}{2}\mathrm{Tr}\left[\Delta\kappa^\dagger - \tilde{\Delta}\tilde{\kappa}^\dagger\right] + T\bar{S}, \tag{S22}$$

where $\kappa, \tilde{\kappa}$ is the pairing tensor for the Nucl+Vap and Vap systems, respectively, and $\Delta_{ij} = \frac{1}{2}\sum_{kl} \bar{v}^{pp}_{ijkl}\kappa_{kl}$ is the corresponding Nucl+Vap pairing field (similar for $\tilde{\Delta}_{ij} = \frac{1}{2}\sum_{kl} \bar{v}^{pp}_{ijkl}\tilde{\kappa}_{kl}$ of the Vap system). Performing variations with both $\rho$ and $\kappa$ leads to the FT-RHB equation for the Nucl+Vap system:

$$\begin{pmatrix} h-\lambda & \Delta \\ -\Delta^* & -h-\lambda \end{pmatrix} \begin{pmatrix} U \\ V \end{pmatrix} = E \begin{pmatrix} U \\ V \end{pmatrix}, \tag{S23}$$

where $(U, V)$ is the q.p. wave function and $E$ the corresponding q.p. energy. On the other hand, by varying the Eq. (S22) with $\tilde{\rho}$ and $\tilde{\kappa}$ we get the FT-RHB equation for the vapor

$$\begin{pmatrix} \tilde{h}-\lambda & \tilde{\Delta} \\ -\tilde{\Delta}^* & -\tilde{h}-\lambda \end{pmatrix} \begin{pmatrix} \tilde{U} \\ \tilde{V} \end{pmatrix} = \tilde{E} \begin{pmatrix} \tilde{U} \\ \tilde{V} \end{pmatrix}, \tag{S24}$$

where $(\tilde{U}, \tilde{V})$ is the vapor system wave function and $\tilde{E}$ is the eigenvalue. The system of equations (S23) and (S24) is coupled by the direct Coulomb term as well as the subsidiary condition on the chemical potential, Eq. (S15). The main difference between the Vap and Nucl+Vap fields is in their initialization for the self-consistent iteration procedure. The Nucl+Vap fields are initialized by the Woods-Saxon potential (both $S$ and $V$) together with

the Coulomb field for the homogeneous charge distribution. On the other hand, the nuclear part of the vapor potentials $\tilde{V}$ and $\tilde{S}$ are taken as vanishing, with only the Coulomb field contribution, having the same strength as for the Nucl+Vap system. We note that during the self-consistent iterations, the nuclear part of the Vap fields acquires some small non-vanishing value. The pairing field $\Delta$ of the Nucl+Vap system is initialized by a constant pairing gap, and same for the Vap system. Independent of the initial pairing strength, we find that the Vap pairing field $\tilde{\Delta}$ is always vanishing.

For the pairing part of the nuclear Hamiltonian we employ the separable interaction, first introduced in Ref. [8], of the form

$$v^{pp}(1,2) = -G\delta\left(\boldsymbol{R}-\boldsymbol{R}'\right)P(\boldsymbol{r})P\left(\boldsymbol{r}'\right), \tag{S25}$$

where $\boldsymbol{R} = 1/2(\boldsymbol{r}_1+\boldsymbol{r}_2)$ and $\boldsymbol{r} = \boldsymbol{r}_1 - \boldsymbol{r}_2$ denote the center-of-mass and relative coordinate, respectively, while $P(\boldsymbol{r})$ has the form

$$P(\boldsymbol{r}) = \frac{1}{\left(4\pi a^2\right)^{3/2}} e^{-\boldsymbol{r}^2/4a^2}. \tag{S26}$$

The parameters $G$ and $a$ used together with the DD-ME2 and DD-PC1 interactions are taken from Ref. [8], while the parameters used with the DD-PCX interaction are from [9]. For the DD-PCJ family of functionals those values can be found in Ref. [10].

### D. Comparison of the relativistic and non-relativistic models without the BLV subtraction

We first compare the relativistic and non-relativistic model calculations using the FT-RHB and finite temperature Hartree–Fock–Bogoliubov (FT-HFB) methods without the BLV procedure. For comparison, the results for the Skyrme-type SkM* interaction are taken from Ref. [11]. Since the FT-HFB results shown in Fig. 8 in Ref. [11] assume spherical symmetry, the FT-RHB calculations are also performed by assuming spherical symmetry to ensure the fairness of the comparison. In Figure 1, the two-neutron separation energies ($S_{2\mathrm{n}}$) and the corresponding chemical potentials ($\lambda_\mathrm{n}$) are displayed for the selected isotopic chains with increasing neutron number and temperature using SkM* (a)-(b) and DD-PC1 (c)-(d) interactions. The $S_{2\mathrm{n}}$ values are calculated using $S_{2\mathrm{n}} = E(Z,N) - E(Z,N-2)$, where $E$ stands for the total binding energy of the nucleus ($E < 0$), and we only present bound

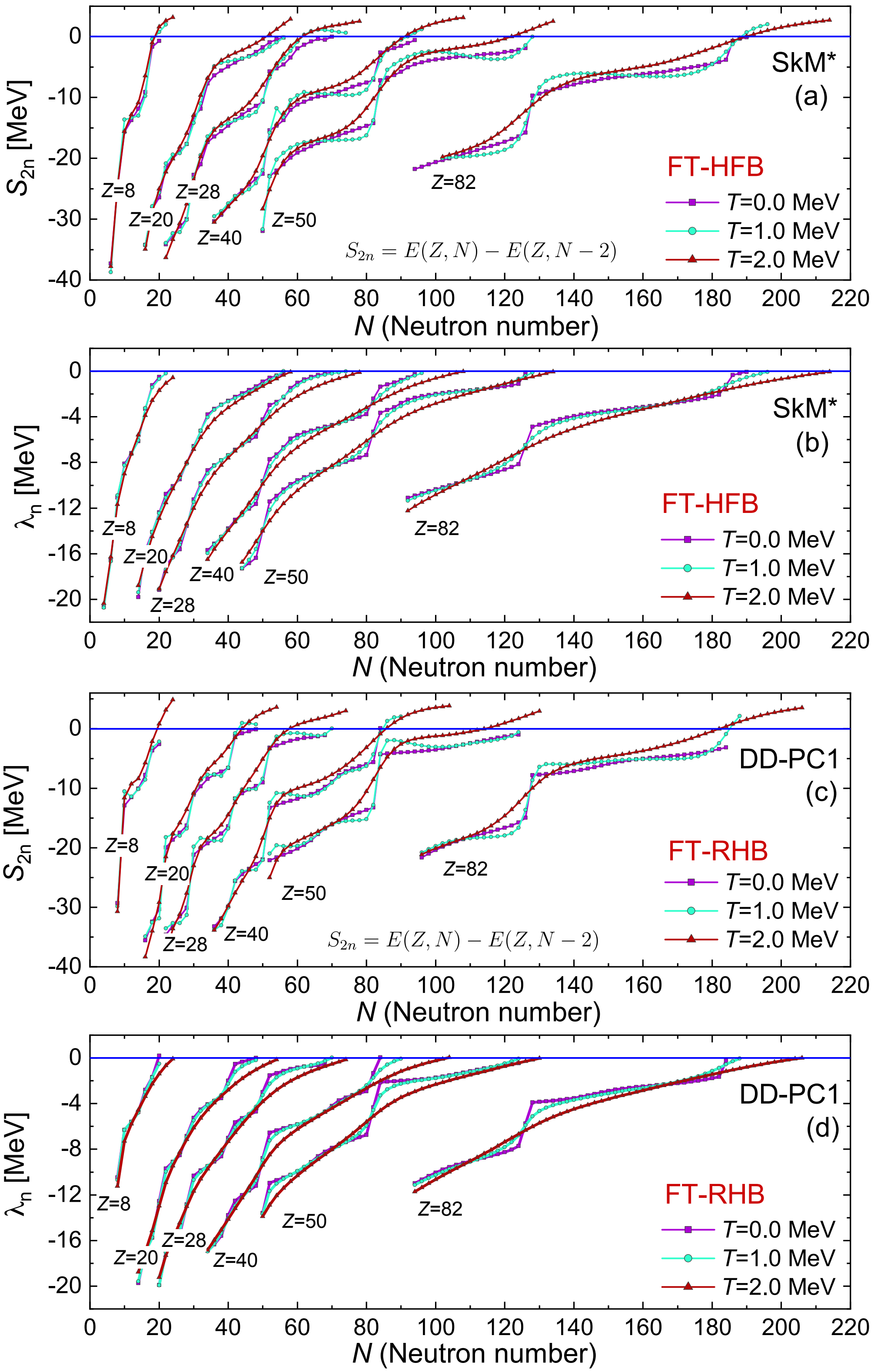


Supplementary Figure 1. Isotopic dependence of two-neutron separation energy $S_{2\mathrm{n}}$ and neutron chemical potential $\lambda_{\mathrm{n}}$ for the selected even-even nuclei using the FT-HFB with SkM* (a)-(b) [11] and FT-RHB with DD-PC1 (c)-(d) interactions at $T = 0, 1.0$ and 2.0 MeV.

nuclei with $\lambda_{\rm n} \leq 0$, which is consistent with the approach in Ref. [11]. It is clear that both relativistic and non-relativistic models predict the same behaviour with increasing neuron number and temperature. At zero temperature, the $S_{2\rm n}$ and $\lambda_{\rm n}$ increase with increasing neutron number, as expected. The sharp changes in the $S_{2\rm n}$ and $\lambda_{\rm n}$ values can be seen around the nuclear magic numbers. By increasing temperature, the shell effects start to disappear, and the changes in the $S_{2\rm n}$ and $\lambda_{\rm n}$ values become smoother. At high temperatures, i.e., at $T = 2.0$ MeV, the number of nuclei with negative chemical potential or bound nuclei increases. On the other hand, the predictions using the $S_{2\rm n}$ values are not consistent with the behaviour of chemical potentials, especially for neutron-rich nuclei near the drip lines. More nuclei become bound when the chemical potentials are considered, but these nuclei also have positive $S_{2\rm n}$ values and are unstable against two-neutron emission. It is known that continuum effects play an important role in the determination of the position of the drip lines already at zero temperature. At high temperatures, i.e., $T > 1$ MeV, the impact of the states in the continuum increases due to the nucleons scattered throughout this region. Therefore, a special caution must be taken in the calculations for weakly-bound nuclei at high temperature. In Supplementary Note 2 C, we show that the inconsistencies between the location of the neutron drip lines using the chemical potential and two-neutron separation energies can be resolved by the inclusion of the BLV prescription in the model calculations, and using the Free energies to calculate the two-neutron separation energies.

## Supplementary Note 2. NUMERICAL METHODS AND TESTS

Most of the calculations in this work are performed by assuming the axially-deformed reflection symmetric nuclei. However, since the axially-deformed calculations are very time-consuming (especially with the explicit treatment of continuum), we initially check our implementations by assuming the spherically-symmetric mean-field at finite temperature. Two FT-RMF solvers that we employ are:

- FT-RMFBSPL - finite-element method (FEM) algorithm which solves the FT-RMF equations by discretizing the wave functions on a B-spline mesh in the coordinate-space. Our code is based on the method presented in Ref. [12]. The basis parameters are: number of finite-elements $N_{\rm fe}$, order of the B-splines $N_{\rm ord}$ and the box-size $R_{\rm box}$.

- FT-RMFHO - solves the FT-RMF equations by expanding the wave functions in a basis of spherical harmonic oscillator (HO) wave functions $R_{nl}(r, b_0)$, characterized by radial and orbital quantum numbers $(n, l)$, oscillator length $b_0$ and maximal number of oscillator shells $N_{\rm osc}$.

These two solvers do not include pairing correlations. However, we use them to perform test calculations for nuclei at temperatures $T \geq 1$ MeV, at which pairing correlations mostly vanish. For calculations where the deformation effects are relevant, we employ the code:

- FT-DIRHBz - solves the axially-deformed FT-RHB equations by assuming reflection symmetry. It employs expansion of the wave functions and fields in the axially-deformed HO basis. The code is based on Ref. [13].

All calculations in this section employ the relativistic DD-ME2 interaction.

### A. Convergence with the increasing box-size

For weakly-bound nuclei, even low temperatures can cause results not to converge with the box-size $R_{\rm box}$ due to the scattering of single-particle states to the continuum. Therefore, we have implemented the BLV subtraction procedure in our calculations. To test the convergence of main observables with increasing $R_{\rm box}$ we select a nucleus in the vicinity of the drip-line, $^{202}$Sm, at $T = 1.0$ MeV. Our finite-temperature calculations in axial-geometry indicate that $^{202}$Sm is in a spherical state at $T = 1$ MeV, with vanishing pairing correlations. We perform calculations with the FT-RMFBSPL code using the DD-ME2 interaction. Results are shown in Fig. 2 for the dependence of entropy $S$ (left panel) and the RMS neutron radius $\sqrt{\langle r_{\rm n}^2\rangle}$ (right panel) on box-size $R_{\rm box}$. This is a drastic example that demonstrates the importance of proper treatment of continuum in drip-line nuclei at finite temperature. In the case where we include the continuum subtraction within the BLV prescription (blue circles), results are independent of the box-size, starting from $R_{\rm box} \approx 20$ fm. If the continuum is not subtracted (red squares), we observe an almost linear increase in entropy and an exponential increase in the RMS neutron radius with increasing box-size.

These results can be readily understood when observing the vector density $\rho_{\rm v}$. In Fig. 3 we display the radial dependence of the total vector density for $R_{\rm box} = 30$ and 40 fm. Imposed box-boundary conditions necessitate that the density has to vanish at $R_{\rm box}$. Due

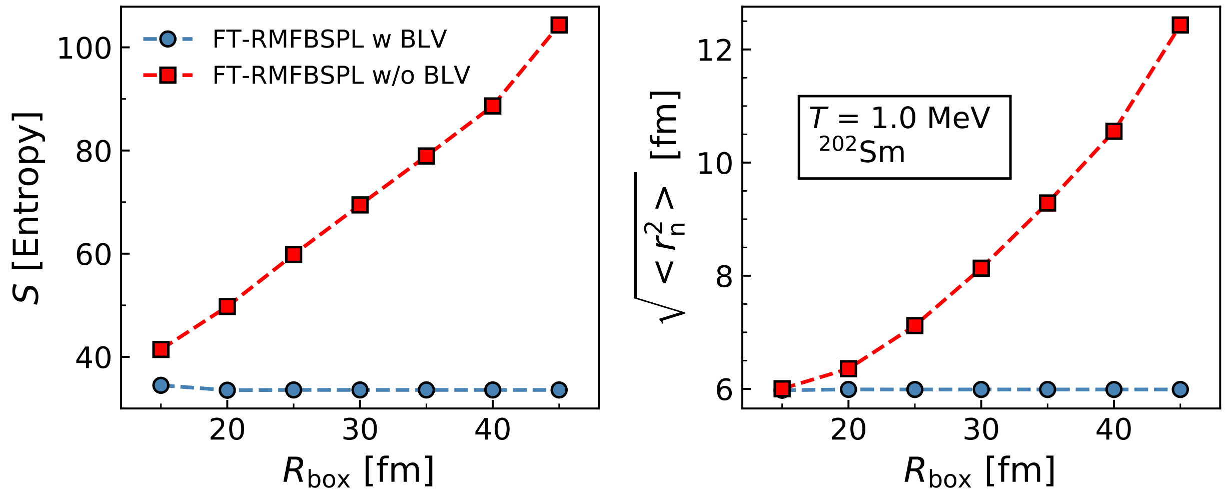


Supplementary Figure 2. The dependence of entropy $S$ (left panel) and RMS neutron radius $\sqrt{\langle r_{\rm n}^2 \rangle}$ (right panel) on box-size $R_{\rm box}$ for $^{202}$Sm at $T = 1$ MeV. Calculations are performed using the FT-RMFBSPL solver with the BLV prescription (blue) and without the BLV prescription (red).

to the contribution of continuum states, without BLV subtraction, one obtains a long tail in density (red dashed line), constant up to $R_{\rm box}$. As the box-size increases so does the density tail. The single-particle states in the continuum, responsible for the long density tail, behave as a vapor that surrounds the nucleus. Such a vapor is precisely the reason for diverging results shown in Fig. 2. Using the BLV procedure allows one to isolate the vapor (Vap) contribution (blue dotted line) from a combined solution of nucleus and vapor (Nucl+Vap). By subtracting the Vap density from the Nucl+Vap density, we obtain the subtracted density (black solid line) that is independent of the box-size.

### B. Comparison between the FT-RMFHO and FT-RMFBSPL solvers

It is well known that the HO basis, due to its Gaussian tail, cannot reproduce the proper asymptotic behavior of the nuclear density [14]. Such a drawback does not pertain to serious issues when studying the nuclei in the vicinity of stability valley, however, it can lead to wrong results for phenomena in weakly-bound nuclei near the drip lines. Such an issue could be resolved by: (i) increasing the number of oscillator shells $N_{\rm osc}$, or (ii) a local-scale transformation [15]. However, the computational time significantly increases with $N_{\rm osc}$, while the local-scale approximation has only been applied at zero-temperature

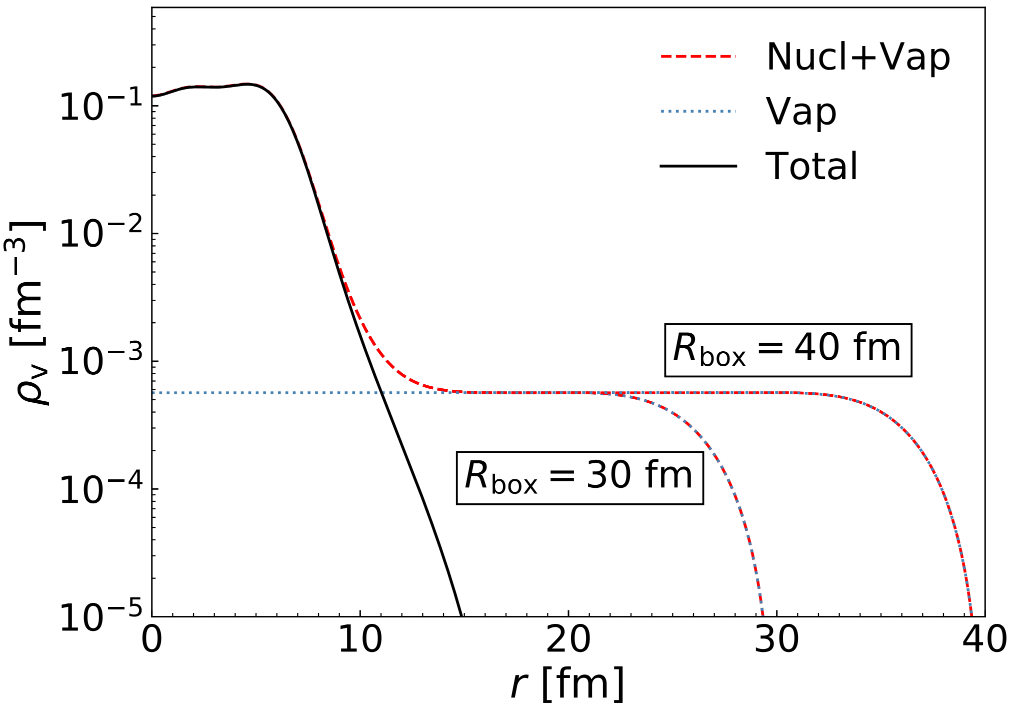


Supplementary Figure 3. Radial dependence of the total vector density $\rho_{\text{v}}$ as calculated with the FT-RMFBSPL solver using the BLV subtraction for $^{202}$Sm at $T = 1$ MeV. Results are shown for $R_{\text{box}} = 30$ and 40 fm. Separately shown are the density contributions of the Nucl+Vap system (red dashed line), the Vap system only (blue dotted line), and the subtracted (total) density (solid black).

for the relativistic EDFs [16]. Even at zero temperature, the transformed HO (THO) basis has significant difficulties for the relativistic functionals, due to contribution of anti-particle states. Later, we will explicitly demonstrate the issue with anti-particles in determining the optimal basis parameters for HO expansion. Therefore, in this subsection we compare the results for important observables at finite temperature obtained with the FT-RMFHO solver that employs the HO expansion to the coordinate-space FT-RMFBSPL solver.

First, we have to select an optimal number of shells $N_{\text{osc}}$ and oscillator length $b_0$ for our comparison. In the case of the HO based solver, the box-size is approximately given by $R_{\text{box}} \approx \sqrt{2N_{\text{osc}}} b_0$ [14]. In the finite-temperature calculations, if the number of shells $N_{\text{osc}}$ is fixed, $b_0$ should be determined as to minimize the subtracted free energy $\bar{F}$. Note that the subtracted free-energy is defined as

$$\bar{F} = F^{Nucl+Vap} - F^{Vap}, \tag{S27}$$

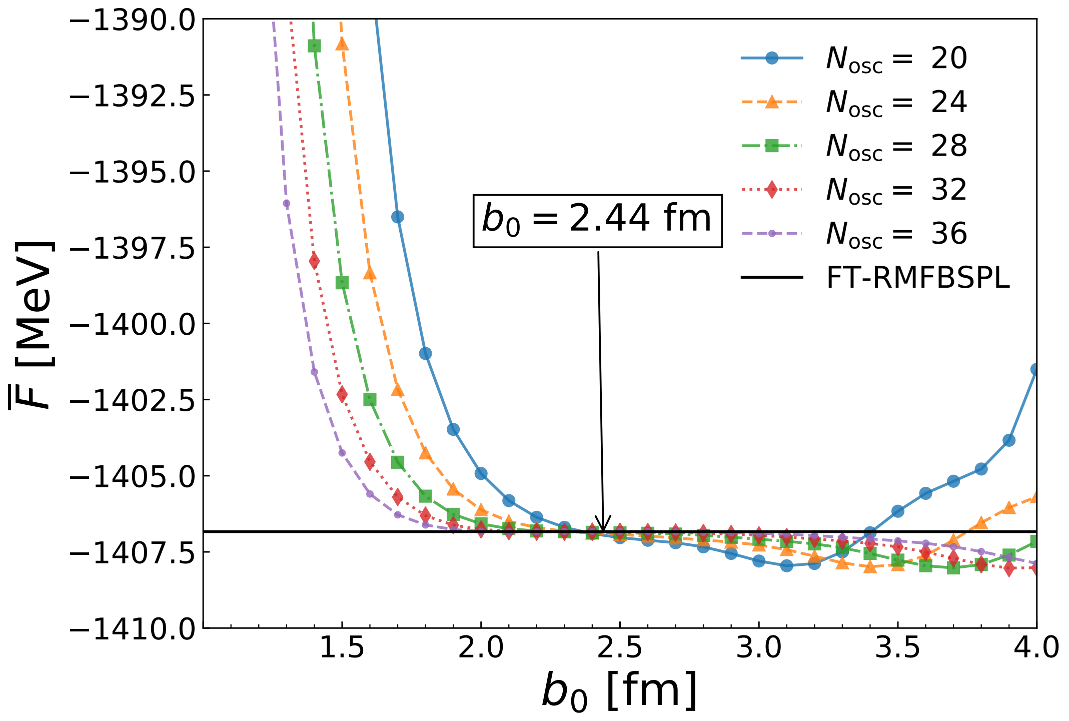


Supplementary Figure 4. The subtracted free energy $\bar{F}$ as a function of the oscillator length $b_0$ for increasing number of HO shells $N_{\rm osc}$. The black horizontal line denotes the FT-RMFBSPL result with $R_{\rm box} = 30$ fm. The arrow indicates the optimal $b_0$ value obtained by setting $\hbar\omega_0 = 41A^{-1/3}$ MeV. Calculations are performed for $^{202}$Sm at $T = 1$ MeV.

where $F^{Nucl+Vap}(F^{Vap})$ is the free energy of the Nucl+Vap(Vap) system. In Figure. 4, we show the $(\bar{F}, b_0)$ plot for changing number of $N_{\rm osc}$ from 20 to 36, for $^{202}$Sm at $T = 1.0$ MeV. Results are compared with the coordinate-space FT-RMFBSPL results (black full line) with $R_{\rm box} = 30$ fm. Results obtained with the FT-RMFHO converge to the FT-RMFBSPL results within a valley of finite width in $b_0$, starting already from $N_{\rm osc} = 20$ shells. We note that the minima obtained at higher values of the oscillator length, $b_0 \gtrsim 3$ fm, are artificial and stem from the anti-particle contribution within the RMF theory. In order to determine the optimal $b_0 = \sqrt{\frac{\hbar}{m\omega_0}}$, we use the mass-dependent formula $\hbar\omega_0 = 41A^{-1/3}$ fm. The result obtained using this formula is indicated by an arrow in Fig. 4 and works well even for $N_{\rm osc} = 20$. We have verified that such a formula for $b_0$ works well across the isotopic chain, for changing temperatures. Therefore, we should expect a good agreement between the results calculated using the FT-RMFBSPL and the FT-RMFHO.

As a test example, we choose samarium isotopic chain ($Z = 62$) at $T = 2.0$ MeV and

compare the results for: subtracted entropy $\bar{S}$, subtracted free energy per nucleon $\bar{F}/A$, neutron root-mean-square (RMS) radii and neutron chemical potential $\lambda_{\rm n}$, between the two codes. At $T = 2.0$ MeV, pairing effects vanish for all nuclei considered in this work, while the deformation effects are significantly reduced. Therefore, imposing the spherical symmetry for this test is justified. Results are shown in Fig. 5(a)-(d), where for the FT-RMFBSPL solver we use a box-size of $R_{\rm box} = 30$ fm, while the FT-RMFHO results are shown for $N_{\rm osc} = 20$ and $N_{\rm osc} = 60$ HO shells both for fermion and boson states. Only the nuclei with negative neutron(proton) chemical potential ($\lambda_{\rm n(p)} < 0$) are shown. It can be inferred that the agreement between the coordinate-space FT-RMFBSPL solver and the FT-RMFHO is excellent, both for $N_{\rm osc} = 20$ and $N_{\rm osc} = 60$ number of oscillator shells.

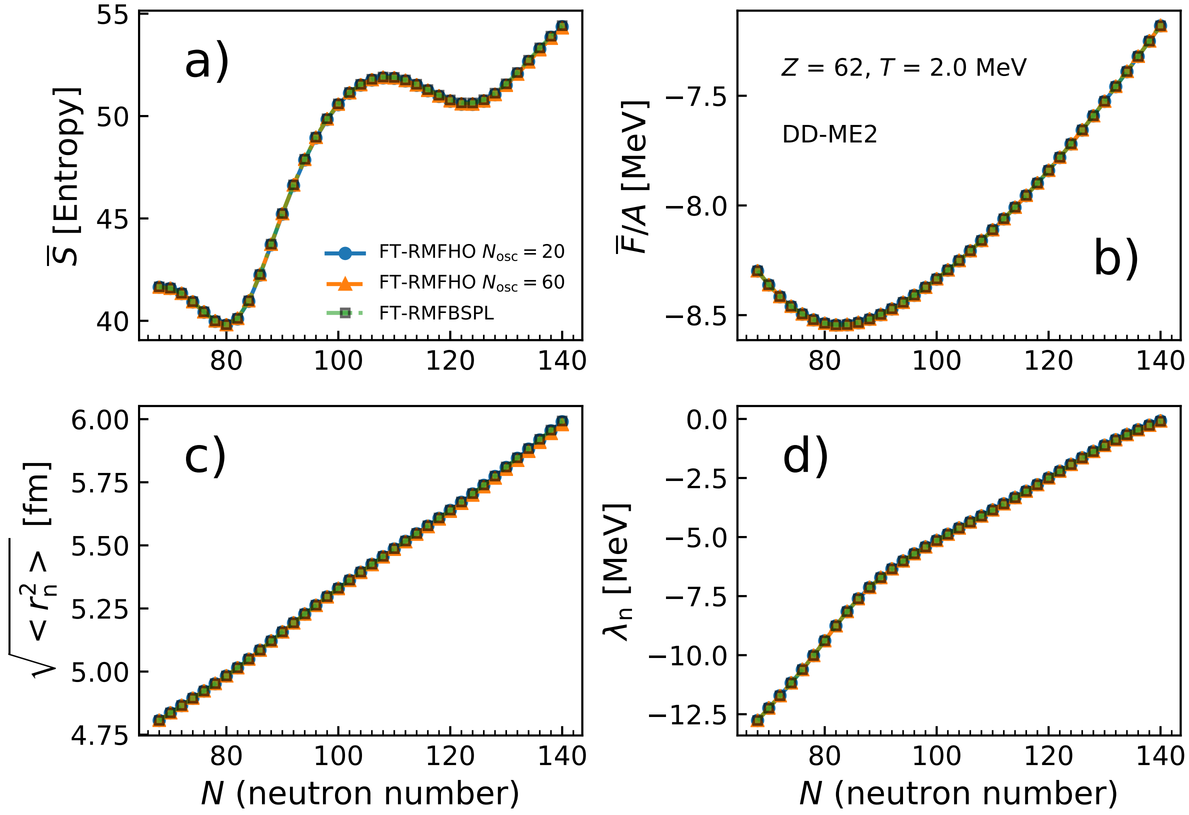


Supplementary Figure 5. Dependence of the subtracted entropy $\bar{S}$ (a), subtracted free energy per nucleon $\bar{F}/A$ (b), RMS neutron radius $\sqrt{\langle r_{\rm n}^2\rangle}$ (c), and neutron chemical potential $\lambda_{\rm n}$ (d) on neutron number for samarium chain ($Z = 62$). Calculations are performed at $T = 2.0$ MeV for DD-ME2 interaction with the FT-RMFHO using $N_{\rm osc} = 20$ (blue) and 60 (orange) oscillator shells and the FT-RMFBSPL with box-radius $R_{\rm box} = 30$ fm (green).

### C. Test of chemical potential

At finite temperatures, the binding energy $E(Z,N)$ should be replaced by the free energy $F(Z,N)$, therefore, the two-neutron and two-proton drip lines are defined by

$$S_{2\mathrm{n}} = F(Z,N) - F(Z,N-2), \quad S_{2\mathrm{p}} = F(Z,N) - F(Z-2,N), \tag{S28}$$

respectively. In the BLV method, the subtracted free-energy $\bar{F}$ is employed. This quantity does not take into account the vapor contribution and can be expressed as $\bar{F}(Z,N) = \bar{E}(Z,N) - T\bar{S}(Z,N)$, where $\bar{E}$ and $\bar{S}$ represent the subtracted total binding energy and entropy, respectively. Figure 6 shows the impact of BLV subtraction on the total energy $E$ and entropy $S$ of the $Z = 82$ isotopic chain at $T = 2$ MeV. The results are presented for nuclei that satisfy the condition $\lambda_{\mathrm{n}} \leq 0$. It is evident that the inclusion of the BLV prescription significantly affects the number of bound nuclei. Furthermore, the difference between the predictions for $E$ and $S$ with and without BLV is particularly pronounced for nuclei near the drip lines. The last bound nucleus at $N = 186$ exhibits a difference of up to 40 MeV (30 MeV) for $E$ ($S$), demonstrating the importance of the BLV subtraction for such systems. Then, it remains to be proved that the two-neutron(proton) drip line defined by $S_{2\mathrm{n}(2\mathrm{p})}$ is equivalent to the one defined by the neutron(proton) chemical potential $\lambda_{\mathrm{n}(\mathrm{p})}$. Although such equivalence at zero-temperature is trivial for even-even nuclei, it is not immediately obvious at finite temperature within the BLV prescription. Our results using the BLV method are displayed in Fig. 7, where for multiple isotopic chains we compare the two-neutron separation energy $S_{2\mathrm{n}}$ (upper panel) and neutron chemical potential $\lambda_{\mathrm{n}}$ (lower panel) with increasing neutron number at $T = 0, 1.0$ and 2.0 MeV. For each isotopic chain, horizontal lines denote the last nucleus in the chain which satisfies $S_{2\mathrm{n}} \leq 0$ and $\lambda_{\mathrm{n}} \leq 0$ condition. We observe that the drip line nucleus predicted by the $S_{2\mathrm{n}}$ condition is also consistent with the drip line nucleus predictions according to the $\lambda_{\mathrm{n}}$ condition. The neutron drip lines calculated by two conditions agree reasonably well with the inclusion of the BLV method, and using the free energies in the calculation of the $S_{2\mathrm{n}(2\mathrm{p})}$ values.

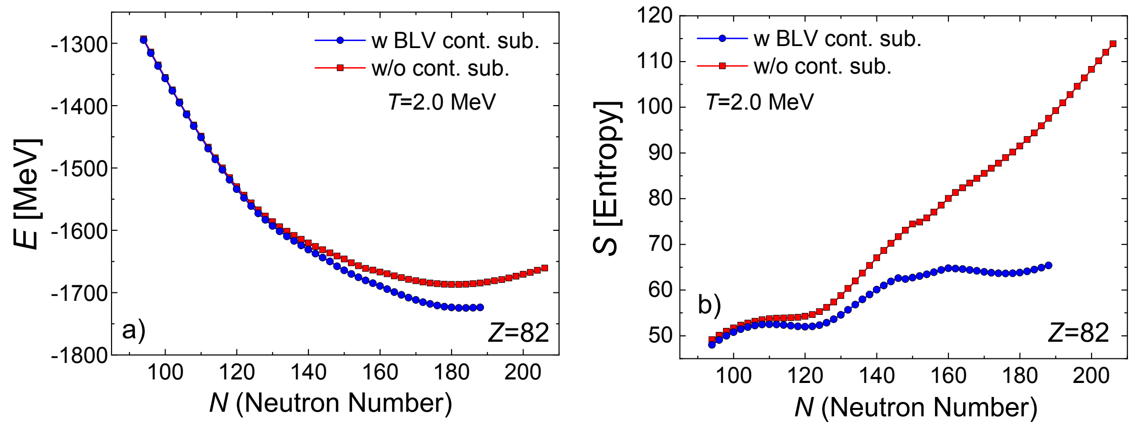


Supplementary Figure 6. The total energy $E$ (a) and entropy $S$ (b) at $T$ =2 MeV as a function of neutron number for Z=82 isotopic chain with and without the inclusion of the BLV subtraction. The calculations are performed using the DD-PC1 interaction.

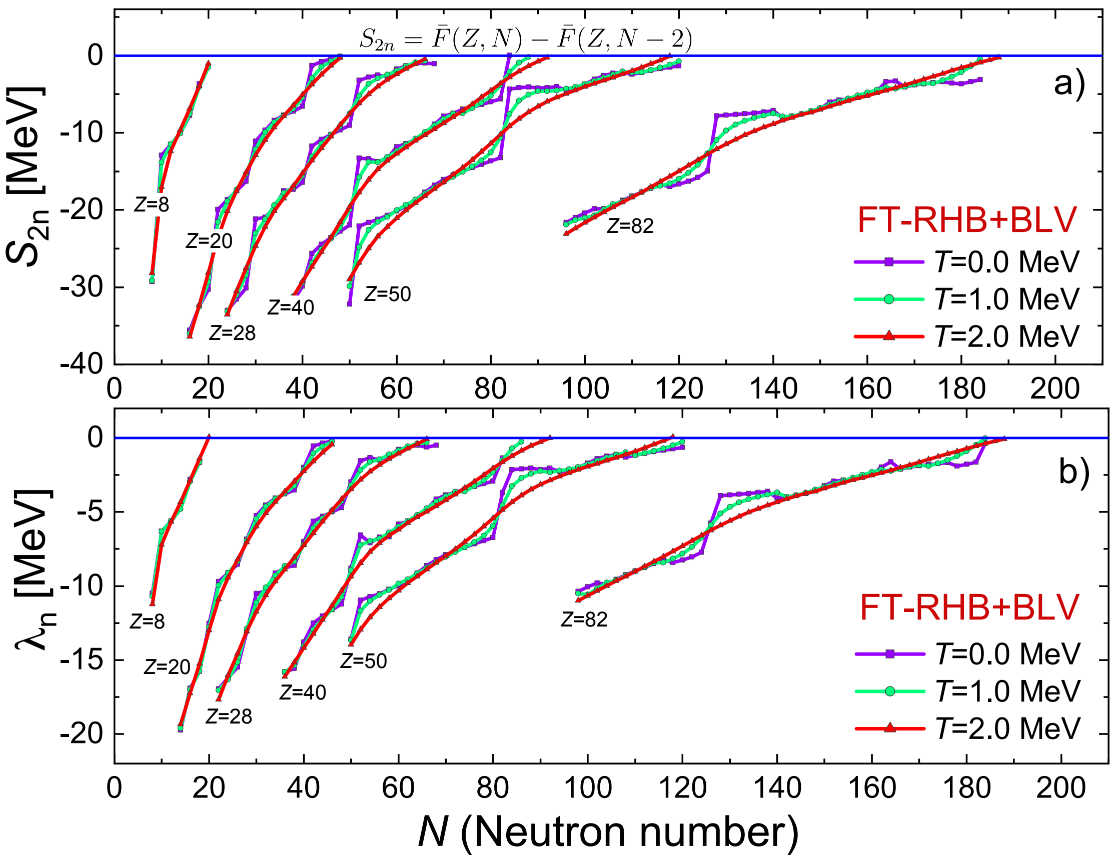


Supplementary Figure 7. Isotopic dependence of two-neutron separation energy $S_{2n}$ (a) and neutron chemical potential $\lambda_n$ (b) for the selected even-even nuclei using using the FT-RHB+BLV method with DD-PC1 interaction.

## Supplementary Note 3. CALCULATION OF NEUTRON EMISSION LIFETIMES

Before presenting the results of neutron emission lifetimes, it is important to benchmark our model against existing theoretical calculations. This is especially relevant to this work because we use the harmonic oscillator basis, which does not reproduce the exponential tail decrease of the density at large distances from the nucleus. To achieve this aim, we calculate the neutron emission lifetimes at finite temperatures using two numerical approaches: (i) discretizing Eqs. (S23) and (S24) in the basis of the harmonic oscillator (FT-RMFHO) and (ii) discretizing in the B-spline basis, corresponding to the solution in the coordinate space (FT-RMFBSPL). Calculations in the coordinate-space basis are known for reproducing the tails of particle density, which is of special significance for the study of weakly-bound systems and neutron halos [14, 17–19]. Unlike being in the ground state at zero temperature, nuclei at finite temperature are found in highly-excited meta-stable states that can decay either by particle emission, provided that the excitation energy is above the particle-decay threshold, or by gamma emission. In particular, as the temperature increases, more and more nuclei gain a finite width for neutron emission. The neutron emission width $\Gamma_\mathrm{n}$ can be obtained from the nucleosynthesis formula [2]

$$\frac{\Gamma_\mathrm{n}}{\hbar} = n_\mathrm{gas}\langle \sigma v \rangle, \tag{S29}$$

where $\sigma$ is the neutron capture cross-section, $\langle v \rangle$ is the average velocity of particles in the external nucleon gas, and $n_\mathrm{gas}$ is the neutron vapor density calculated as number of neutrons in the vapor divided by the discretization volume. The neutron cross-section can be approximated by the geometric cross section $\sigma = \pi R^2$ [2], where the RMS-radius $R$ is obtained from the FT-RHB calculations. Finally, the neutron emission lifetime can be calculated as $\tau_\mathrm{n} = \hbar/\Gamma_\mathrm{n}$. The statistical velocity is calculated from the finite-temperature canonical single-particle neutron energies $\varepsilon_\mathrm{n}$ assuming the Fermi-Dirac distribution of neutrons $f(\varepsilon_\mathrm{n})$, therefore [20]

$$\langle v \rangle = \frac{\int\limits_0^\infty f(\varepsilon_\mathrm{n}) v(\varepsilon_\mathrm{n}) \sqrt{\varepsilon_\mathrm{n}} d\varepsilon_\mathrm{n}}{\int\limits_0^\infty f(\varepsilon_\mathrm{n}) \sqrt{\varepsilon_\mathrm{n}} d\varepsilon_\mathrm{n}}, \quad v(\varepsilon_\mathrm{n}) = \sqrt{\frac{2\varepsilon_\mathrm{n}}{m_\mathrm{n}}}, \tag{S30}$$

where $m_\mathrm{n}$ is the neutron mass. We obtain the canonical single-particle neutron vapor states by diagonalizing the neutron vapor density $\tilde{\rho}_\mathrm{n}$ and transforming the corresponding quasi-

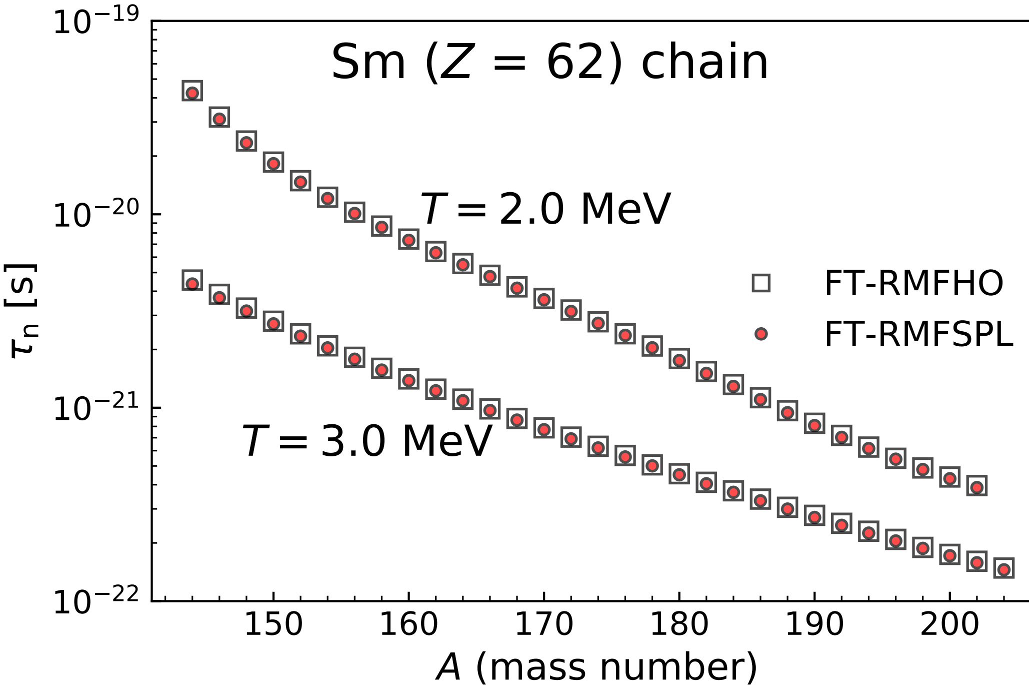


Supplementary Figure 8. Isotopic dependence of neutron emission lifetimes $\tau_{\rm n}$ for samarium ($Z = 62$) chain at $T = 2$ and 3 MeV. Results are calculated by either expansion in the basis of the harmonic oscillator (FT-RMFHO) or discretization in the coordinate-space (FT-RMFBSPL), using the relativistic DD-ME2 interaction.

particle energies in this basis. Since we lose the notion of independent quasi-particles at finite-temperature, this procedure is an approximation.

To check if the HO basis provides reasonable results in comparison to coordinate-space-based methods, we choose large temperatures of $T = 2$ and 3 MeV and assume that all nuclei are spherical and in a normal state (no pairing correlations), which allows for detailed calculations. As we will demonstrate in Supplementary Note 4 such an assumption is reasonable. Reducing the basis size due to spherical degeneracy is especially significant for the coordinate-space code, where the calculation time becomes prohibiting for systematic deformed calculations. High temperatures are chosen to exaggerate the influence of the particle continuum. Calculations are performed for the samarium ($Z = 62$) isotopic chain. The FT-RMFHO code is characterized by the number of oscillator shells $N_{\rm osc}$ in which the single-particle basis is expanded, while the wave functions of the coordinate-space code FT-RMFBSPL are discretized within a radius $R_{\rm box}$. For this test we set $R_{\rm box} = 20$ fm. Since

Supplementary Table 1. The neutron emission widths $\Gamma_{\rm n}$ and density of neutron vapor $n_{\rm gas}$ for $^{238}$U and $^{258}$U as calculated with our model (FT-DIRHBz) using the relativistic DD-ME2 interaction, compared to results in Ref. [20].

| | Ref. [20] | | FT-DIRHBz | |
|---|---|---|---|---|
| $^{238}$U, $k_{\rm B}T$ [MeV] | $n_{\rm gas}$ [fm$^{-3}$] | $\Gamma$ [MeV] | $n_{\rm gas}$ [fm$^{-3}$] | $\Gamma$ [MeV] |
| 1.0 | $2.07 \times 10^{-6}$ | $3.69 \times 10^{-3}$ | $1.05 \times 10^{-6}$ | $1.56 \times 10^{-3}$ |
| 1.5 | $2.09 \times 10^{-5}$ | $4.57 \times 10^{-2}$ | $1.30 \times 10^{-5}$ | $2.32 \times 10^{-2}$ |
| 2.0 | $7.67 \times 10^{-5}$ | $1.94 \times 10^{-1}$ | $5.27 \times 10^{-5}$ | $1.07 \times 10^{-1}$ |
| $^{258}$U, $k_{\rm B}T$ [MeV] | $n_{\rm gas}$ [fm$^{-3}$] | $\Gamma$ [MeV] | $n_{\rm gas}$ [fm$^{-3}$] | $\Gamma$ [MeV] |
| 1.0 | $1.67 \times 10^{-5}$ | $3.16 \times 10^{-2}$ | $7.15 \times 10^{-6}$ | $1.13 \times 10^{-2}$ |
| 1.5 | $7.82 \times 10^{-5}$ | $1.82 \times 10^{-1}$ | $4.78 \times 10^{-5}$ | $8.97 \times 10^{-2}$ |
| 2.0 | $2.11 \times 10^{-4}$ | $5.70 \times 10^{-1}$ | $1.49 \times 10^{-4}$ | $3.18 \times 10^{-1}$ |

we set $N_{\rm osc} = 20$, the oscillator length is chosen as $b_0 = 3.0$ fm, to approximately reproduce the $R_{\rm box}$ of the coordinate-space solver. All calculations employ the relativistic DD-ME2 interaction. In Fig. 8 we show the isotopic dependence of neutron emission lifetimes for samarium isotopes at $T = 2$ and 3 MeV. We observe that the agreement between the two calculation methods is excellent, all up to the neutron drip line. Furthermore, the influence of the particle continuum is especially pronounced at $T = 3$ MeV, reflected by more than an order of magnitude shorter neutron emission lifetimes for considered nuclei compared to $T = 2$ MeV. However, this example demonstrates that when supplemented with a proper continuum subtraction procedure, one can use the HO code to study neutron lifetimes within the scope of validity of Eq. (S29).

To further exemplify the validity of our approach, it is instructive to compare the results between different theoretical models. Here we compare our results with those from Ref. [20], which assume axially-deformed nuclei calculated with coordinate-space discretization and non-relativistic Skyrme SLy4 interaction. To this aim, we employ the axially-deformed FT-DIRHBz code with the DD-ME2 interaction and perform calculations for $^{238}$U and $^{258}$U at $T = 1.0, 1.5$ and 2 MeV. Our results are compared with corresponding results from Ref. [20] in Table 1. We observe that the neutron emission widths $\Gamma_{\rm n}$ are consistent between both calculations, being of the same order of magnitude. For $^{238}$U, as the temperature increases,

the number of states in the continuum grows, thus increasing the neutron vapor density $n_{\rm gas}$. Following Eq. (S29), the density of neutron vapor states is proportional to neutron emission widths, and therefore, as the temperature increases, neutron emission lifetimes get shorter. Additional 20 neutrons in $^{258}$U allow for more loosely-bound nucleons contributing to the neutron vapor thus increasing the neutron widths and decreasing the lifetimes for a fixed temperature.

We note the peculiarity in calculating the box radius $R_{\rm box}$ within the axially-deformed HO code. The oscillator lengths are now represented by corresponding oscillator length in radial $b_\rho$ and $z$-direction $b_z$, determined from the volume conservation relation $b_0^3 = b_\rho^2 b_z$, so that $R_{\rm box} \approx \sqrt{2N_{\rm osc}} b_0$. Therefore, within the FT-DIRHBz, the discretization volume is still $4\pi/3 R_{\rm box}^3$.

## Supplementary Note 4. TEMPERATURE EVOLUTION OF QUADRUPOLE DEFORMATION

Within the BLV subtraction procedure, the mean value of an observable $\langle \mathcal{O}[\bar{\rho}] \rangle_T$ at temperature $T$, is a function of the subtracted density ($\bar{\rho}$), defined as the difference between the density of the Nucl+Vap system ($\rho$) and Vap system ($\tilde{\rho}$). For the relativistic EDFs, the baryon density is equal to the vector density $\bar{\rho}_{\rm v}$, which satisfies Eq. (S15). In the following, we present the results for the temperature evolution of isoscalar quadrupole deformation for selected even-even nuclei.

Starting from the proton(neutron) subtracted vector density $\bar{\rho}_v^{\rm p(n)}$ the proton(neutron) quadrupole moment is defined as

$$Q_{20}^{\rm p(n)} = \int d^3 r \bar{\rho}_v^{\rm p(n)}(\boldsymbol{r})(2z^2 - r_\perp^2), \tag{S31}$$

where $(r_\perp, z)$ are the cylindrical coordinates. It is more customary to express the results in terms of dimensionless variable $\beta_2^{\rm p(n)}$ defined as [21]

$$\beta_2^{\rm p(n)} = \frac{1}{2}\sqrt{\frac{5}{4\pi}} \frac{3}{4\pi} Z(N) R_0^2 Q_{20}^{\rm p(n)}, \tag{S32}$$

where $Z(N)$ denotes the proton(neutron) number and $R_0 = 1.2A^{1/3}$ fm. The isoscalar quadrupole deformation is defined as $\beta_2^{IS} = \beta_2^{\rm p} + \beta_2^{\rm n}$. To demonstrate the effect of the shape phase-transition in more detail, we show the temperature evolution of $\beta_2^{\rm IS}$ for particular

isotopes of neodymium ($Z = 60$) in Fig. 9(a)–(d), as calculated with three relativistic EDFs. For $^{150}$Nd in Fig. 9(a) and DD-ME2 functional, we observe that at low temperatures, deformation can be slightly increased with increasing temperature. For $T > 0.5$ MeV, $\beta_2^{\rm IS}$ decreases smoothly with temperature up to $T_{\rm c}^{\rm p} = 2$ MeV, where it suddenly vanishes. The trends predicted with point-coupling functionals are similar, although $T_{\rm c}^{\rm p} = 2.2$ MeV for DD-PC1 and $T_{\rm c}^{\rm p} = 2.4$ MeV for DD-PCX. A higher phase-transition temperature is predicted with all functionals for $^{170}$Nd in Fig. 9(b) where $_{\rm c}^{\rm p}$ is close to 3 MeV. $^{180}$Nd also has a prolate shape, which reduces to spherical at $_{\rm c}^{\rm p} = 2.4$ MeV for DD-ME2 , $_{\rm c}^{\rm p} = 2.5$ MeV for DD-PC1 and $_{\rm c}^{\rm p} = 2.6$ MeV for DD-PCX. For $^{190}$Nd in Fig. 9(d), the shape phase-transition temperature is lower, being around 1 MeV for all functionals. However, the DD-ME2 predicts a slightly prolate shape of the nucleus, while both DD-PC1 and DD-PCX predict oblate configurations. Note that in the case of $^{190}$Nd the ground-state difference between the oblate and prolate shapes is around 3 MeV, while for $^{150}$Nd it is only around 0.1 MeV, signifying a strong configuration mixing.

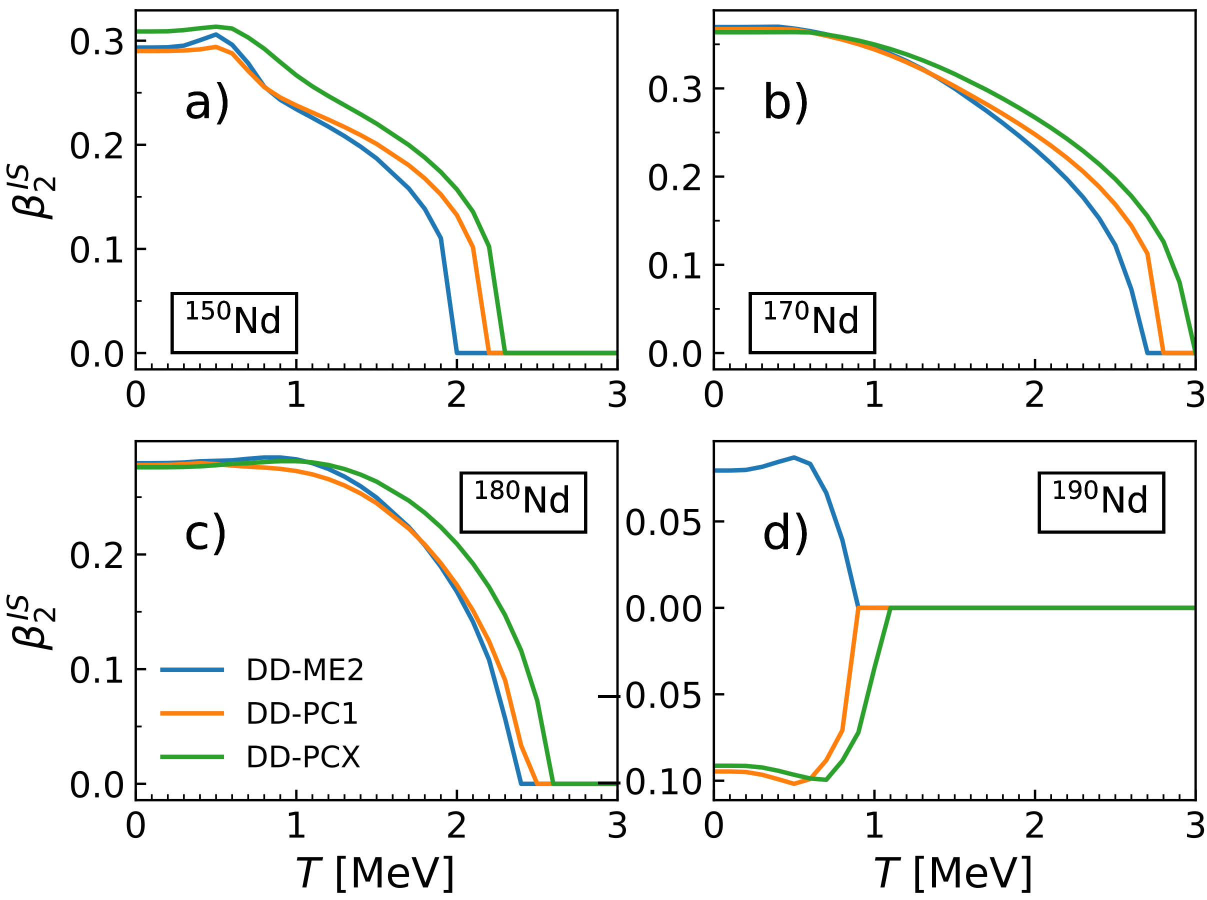


Supplementary Figure 9. Evolution of the isoscalar quadrupole deformation $\beta_2^{\rm IS}$ as a function of the temperature $T$ for $^{150}$Nd (a), $^{170}$Nd (b), $^{180}$Nd (c), and $^{190}$Nd (d). Calculations are performed with DD-ME2 (solid blue), DD-PC1 (solid orange) and DD-PCX (solid green) relativistic EDFs.

## Supplementary Note 5. SYSTEMATIC CALCULATIONS WITH DIFFERENT EDFS

To estimate the systematic uncertainties in predicting the two-nucleon drip lines at finite temperatures, it is instructive to compare results obtained using various EDF parametrizations. In Figure 10 (a)-(h), we show the two-nucleon drip lines calculated with three relativistic EDFs: DD-ME2 (blue), DD-PC1 (red) and DD-PCX (orange) at $T = 0$, 0.5, 0.8, 1.0, 1.2, 1.5, 1.8, and 2.0 MeV. Results for the two-proton drip line agree reasonably well between different functionals. Therefore, we focus on the differences in the two-neutron drip line. At zero temperature, moderate differences are visible between the employed functionals [see Fig. 10(a)]. These differences arise due to the different formulations of the EDF and optimization procedures employed to constrain the underlying model parameters. In general, the DD-PCX tends to predict the two-neutron drip line at a lower number of neutrons $N$. The two-neutron drip lines calculated with the DD-ME2 and DD-PC1 functionals tend to be similar up to $Z \sim 82$. For $Z > 82$ two-neutron drip line predicted by the DD-PCX and DD-ME2 is consistent, while the DD-PC1 predicts more neutron-rich nuclei within the drip line. By increasing temperature slightly, at $T = 0.5$ MeV, the pairing effects decrease considerably for the majority of nuclei in the nuclear landscape, whereas we do not observe a major change in the two-proton and two-neutron drip lines. At $T = 1.0$ MeV [see Fig. 10(d)], pairing correlations have mostly vanished, and shell structure around $N = 126$ and $N = 184$ is almost washed out. Furthermore, the two-neutron drip line becomes smoother around the neutron shell closure. As can be seen in Fig. 10(h), two-neutron drip line with all three functionals assumes almost a linear form at $T = 2.0$ MeV. However, the slopes of these lines are slightly different due to the different structure of the mean-field among functionals as mentioned above. To further elaborate on the differences between functionals, in Tab. 2, we provide the number of even-even nuclei within the drip lines at finite temperatures. If $N_{\text{nucl}}$ denotes the number of even-even nuclei between the drip lines, then it can be inferred that $N_{\text{nucl}}(\text{DD-PC1}) \gtrsim N_{\text{nucl}}(\text{DD-ME2}) > N_{\text{nucl}}(\text{DD-PCX})$, for all studied temperatures. For instance, at $T = 0$ MeV, the DD-ME2 functional predicts 58 bound even-even nuclei more compared to the DD-PCX. By increasing temperature, each functional predicts more bound nuclei between two-proton and two-neutron drip lines. At $T = 2$ MeV, the DD-ME2 functional predicts roughly 100 even-even nuclei more compared to the DD-PCX, which will decay by the equilibrated nucleon evaporation (nuclei between the drip lines). The main

Supplementary Table 2. Number of the bound even-even nuclei between the two-proton and two-neutron drip line in the range $8 \leq Z \leq 104$, for DD-ME2, DD-PC1 and DD-PCX functionals at temperatures $T = 0, 0.5, 0.8, 1.0, 1.2, 1.5, 1.8$ and 2.0 MeV.

| $T$ [MeV] | DD-ME2 | DD-PC1 | DD-PCX |
|---|---|---|---|
| 0.0 | 1623 | 1671 | 1565 |
| 0.5 | 1618 | 1673 | 1562 |
| 0.8 | 1647 | 1687 | 1568 |
| 1.0 | 1655 | 1695 | 1572 |
| 1.2 | 1664 | 1699 | 1579 |
| 1.5 | 1669 | 1702 | 1580 |
| 1.8 | 1678 | 1713 | 1583 |
| 2.0 | 1681 | 1716 | 1588 |

reason for these differences comes from the underlying nuclear matter properties. In Supplementary Note 7, we present calculations with the point-coupling functionals constrained to different symmetry energy at saturation density ($J$) of the symmetric nuclear matter. Since the DD-PCX has lower $J$ compared to both DD-ME2 and DD-PC1, the predicted two-neutron drip line is less neutron-rich. Overall, the number of even-even nuclei between the drip lines at $T = 2$ MeV, compared to zero-temperature, increases by 58 for DD-ME2, 45 for DD-PC1, and 23 for DD-PCX functional. The DD-PCX two-neutron drip line is again less neutron-rich compared to the DD-ME2 and DD-PC1 drip lines, which predict more comparable drip line.

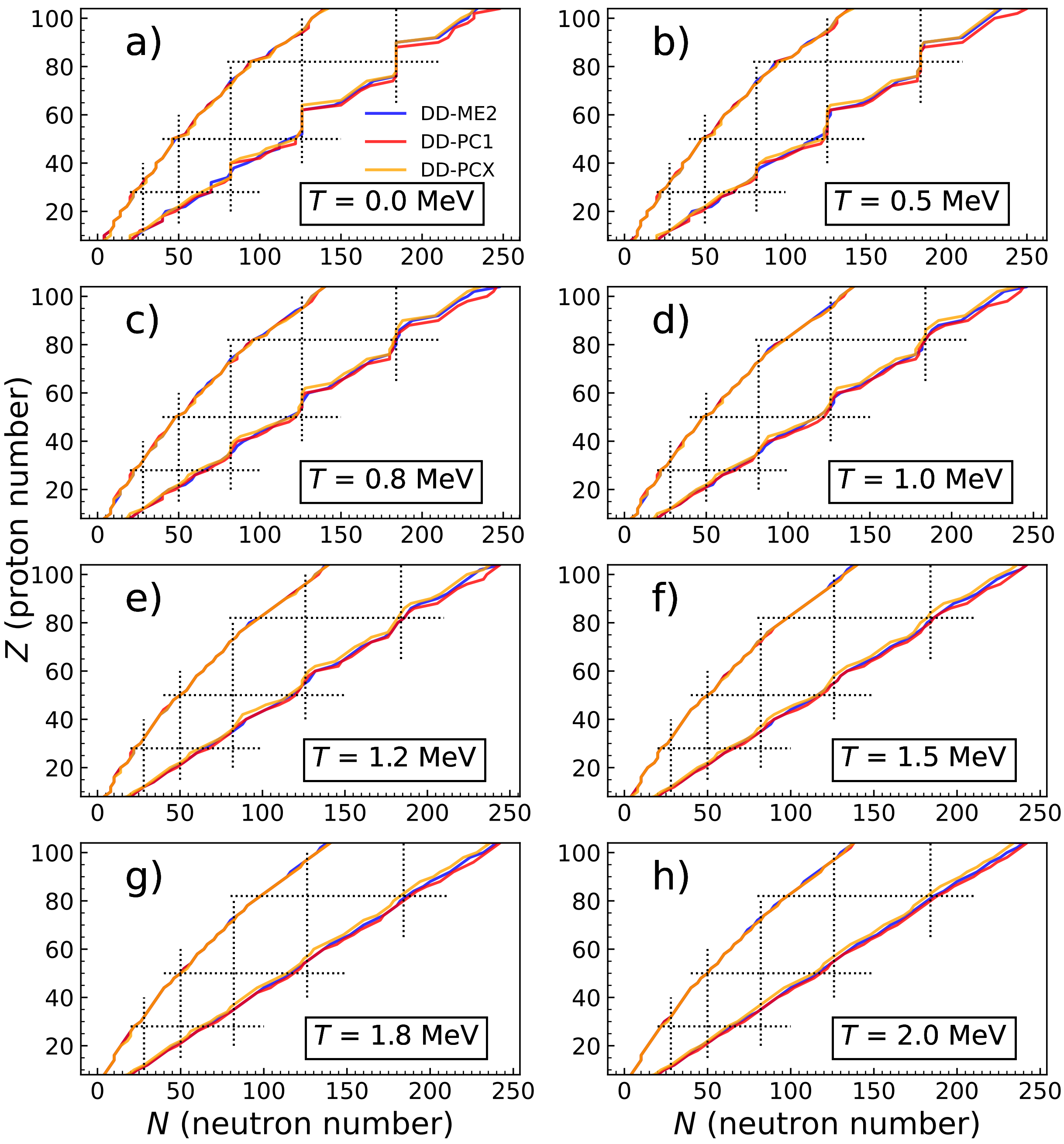


Supplementary Figure 10. (a)-(h) Two-neutron and two-proton drip lines calculated at $T = 0, 0.5, 0.8, 1.0, 1.2, 1.5, 1.8$ and 2.0 MeV using three different parameterizations of the relativistic EDF: DD-ME2 (blue), DD-PC1 (red) and DD-PCX (orange). The vertical and horizontal dotted lines represent the proton and neutron magic numbers.

## Supplementary Note 6. ESTIMATING SYSTEMATIC AND STATISTICAL UNCERTAINTIES

Systematic uncertainties arise from different assumptions used in the underlying EDF interactions [22–24]. For instance, the meson-exchange DD-ME2 interaction consists of three meson-nucleon vertices in its mean-field part, corresponding to the exchange of the $\sigma, \omega$, and $\rho$ mesons, while the point-coupling interactions (DD-PC1 and DD-PCX) reduce the meson propagators to simple delta functions. Following Ref. [21], the systematic uncertainty is given as a spread across different EDFs. Assuming that $A(\boldsymbol{X})$ is an observable of interest, being a function of different variables $\boldsymbol{X}$, we define the systematic uncertainty of observable $A$ as

$$\Delta A(\boldsymbol{X}) = |A_{\max}(\boldsymbol{X}) - A_{\min}(\boldsymbol{X})|, \tag{S33}$$

where $A_{\max(\min)}(\boldsymbol{X})$ represents the maximum(minimum) values of $A(\boldsymbol{X})$ with different EDFs. Note that variables $\boldsymbol{X}$ here represent input parameters of the model, e.g., proton and neutron number or temperature $T$, $\boldsymbol{X} = (Z, N, T, \ldots)$. We take the mean value as the average across different model predictions

$$\bar{A}(\boldsymbol{X}) = \sum_i A^{(i)}(\boldsymbol{X}), \tag{S34}$$

where the sum goes over the DD-ME2, DD-PC1, and DD-PCX interactions.

Statistical uncertainties stem from errors in optimizing the EDF parameters. For their calculation, one requires the so-called covariance matrix, in addition to the first derivative of the observable with respect to the model parameters [22]. In this work, we calculate the statistical errors for the recently optimized functional DD-PCX [9]. Assuming a linear behavior of the observable with small changes in model parameters in the vicinity of the $\chi^2$-optimum, we can calculate the first derivatives either by (i) linear regression or (ii) finite-element derivatives. To this aim, we use the mesh as defined by the second-order Richardson extrapolation [23]. For a given step size $h$ and observable $A(\boldsymbol{p}_0)$, where $\boldsymbol{p}_0$ is the optimal parameter set, it requires evaluation of the observable at 6 points in the parameter space. Assuming $N_p$ parameters, variation of parameter $p_j$ requires calculating:

$$A(p_1, \ldots, p_i', \ldots, p_{N_p}), \quad \text{where} \quad p_i' \in \{p_i \pm h, p_i \pm h/2, p_i \pm h/4\}\,. \tag{S35}$$

Such calculations are very time-consuming, therefore we restrict our interest to the $Z = 60$ isotopic chain. Observable for which we determine statistical uncertainty is the two-neutron

separation energy $A \equiv S_{2n}$, defined in Eq. (S28). The statistical uncertainty $\overline{\Delta A^2}$ around the predicted value $A(\boldsymbol{p}_0)$ is calculated as [22]

$$\overline{\Delta A^2} = \sum_{\alpha\beta} G^A_\alpha \hat{C}_{\alpha\beta} G^A_\beta, \tag{S36}$$

where $\hat{C}$ is the covariance matrix, and $\boldsymbol{G}^A$ is the matrix of the first derivatives of observable $A(\boldsymbol{p}_0)$, i.e., $\boldsymbol{G}^A = \boldsymbol{\partial_p} A|_{\boldsymbol{p_0}}$. In the case of the DD-PCX functional the parameter space consists of $N_\mathrm{p} = 12$ variables: 4 scalar couplings $a_\mathrm{S}, b_\mathrm{S}, c_\mathrm{S}, d_\mathrm{S}$, 3 vector $a_\mathrm{V}, b_\mathrm{V}, d_\mathrm{V}$, 2 isovector $b_\mathrm{TV}, d_\mathrm{TV}$, a derivative-coupling term $\delta_\mathrm{S}$ and 2 pairing strength parameters for protons and neutrons $G_\mathrm{p}, G_\mathrm{n}$. Therefore, covariance matrix is a $12 \times 12$-dimensional with 12-dimensional derivative column vectors $\boldsymbol{G}^A$. In Figure 11, we show the statistical uncertainties of the neodymium ($Z = 60$) chain at $T = 1$ MeV calculated by either using the linear regression (method i) or the Richardson extrapolation formula (method ii) to estimate the derivative matrix $\boldsymbol{G}^A$. As can be seen from the figure, both methods produce consistent results, providing an additional check for our calculation. Overall, statistical errors are small, being less than 1 MeV.

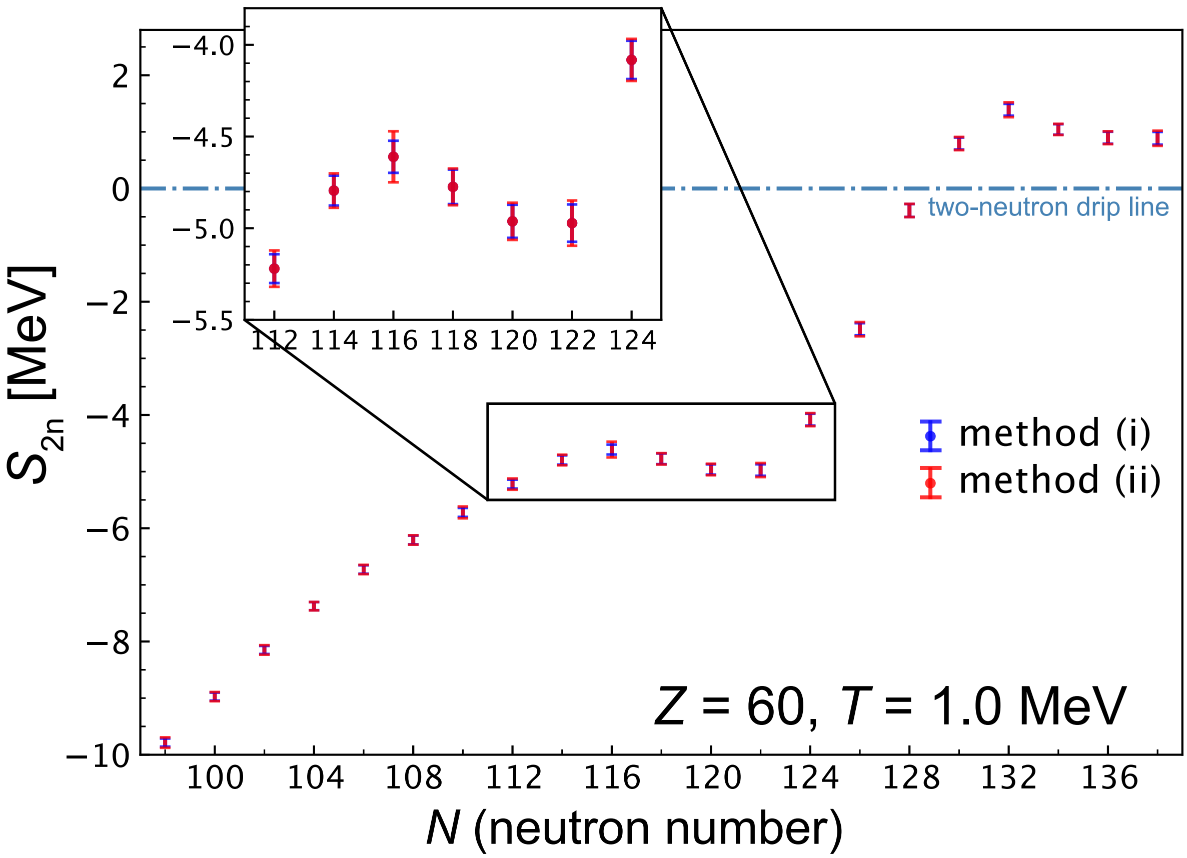


Supplementary Figure 11. Statistical uncertainties for the DD-PCX interaction in two-neutron separation energy $S_{2n}$ for the neodymium chain ($Z = 60$) at $T = 1$ MeV. The derivative matrix $\boldsymbol{G}^A$ in Eq. (S36) is calculated either using method (i) corresponding to linear regression (blue) or method (ii) which uses the Richardson extrapolation formula (red). The inset shows an enlarged region between $N = 112$–$124$ to better visualize the differences.

## Supplementary Note 7. DEPENDENCE OF DRIP LINES ON THE SYMMETRY ENERGY

Concerning the discussion on the dependence of the drip lines on the symmetry energy at the saturation density $J$, related to Figure 3 in the main manuscript, here we calculate the temperature dependence of two-proton and two-neutron drip lines by employing the functionals DD-PCJ30, DD-PCJ32, DD-PCJ34 and DD-PCJ36 for temperatures $T = 0, 0.5, 1, 1.5$ and 2 MeV. Results are shown in Fig. 12(a)–(e). For all considered temperatures, the functionals with higher $J$ predict the existence of more neutron-rich nuclei compared to those with lower $J$. Such a conclusion is plausible since a higher value of the symmetry energy favours a larger neutron skin thickness, thus favouring more neutron-rich systems [25, 26]. If we focus on the effect of finite temperature on the drip line, irrespective of the employed functional, the conclusion is the same — increasing the temperature leads to the vanishing of the nuclear shell effects. The results shown in table 2, which demonstrated that $N_{\text{nucl}}(\text{DD-PC1}) \gtrsim N_{\text{nucl}}(\text{DD-ME2}) > N_{\text{nucl}}(\text{DD-PCX})$, for all considered temperatures, are in agreement with these considerations. Considering DD-PC1 ($J = 33$ MeV), DD-ME2 ($J = 32.3$ MeV), and DD-PCX ($J = 31.3$ MeV) functionals, we find that a similar hierarchy as in DD-PCJ family with symmetry energy at saturation density $J$ is found. However, DD-PCJ functionals are optimized using the same set of finite-nuclei and nuclear matter properties with only $J$ varied. On the other hand, DD-PC1, DD-PCX and DD-ME2 considerably differ in their optimization protocols and employed nuclear properties. Therefore, although our results show that those functionals follow similar trends as DD-PCJ set, detailed assessment of the correlation of $N_{\text{nucl}}$ with $J$ requires investigation of the density dependence of symmetry energy $S_2(\rho)$.

The total number of bound nuclei $N_{\text{nucl}}$ is shown in Fig. 13 as a function of temperature, and for different point-coupling functionals (DD-PCJ family). A clear hierarchy is noticed, where the largest number of bound nuclei is predicted by the DD-PCJ36, and least for DD-PCJ30. Such hierarchy holds for all considered temperatures. Furthermore, all functionals predict an increase in number of bound nuclei above $T > 1$ MeV.

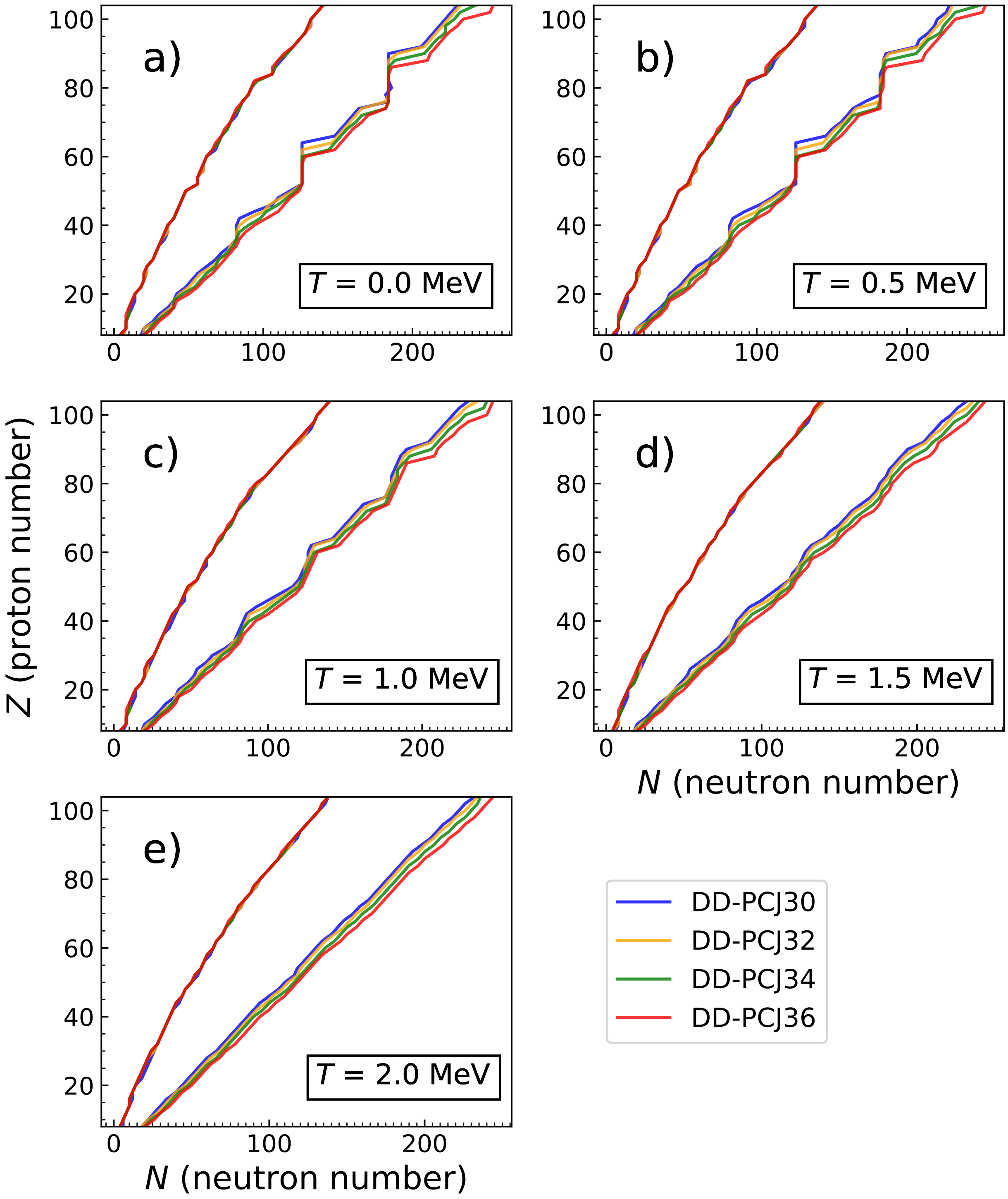


Supplementary Figure 12. (a)–(e) The temperature evolution of two-nucleon drip lines at $T = 0, 0.5, 1, 1.5$ and 2 MeV as calculated with the density-dependent point-coupling functionals constrained to $J = 30, 32, 34$ and 36 MeV symmetry energy values.

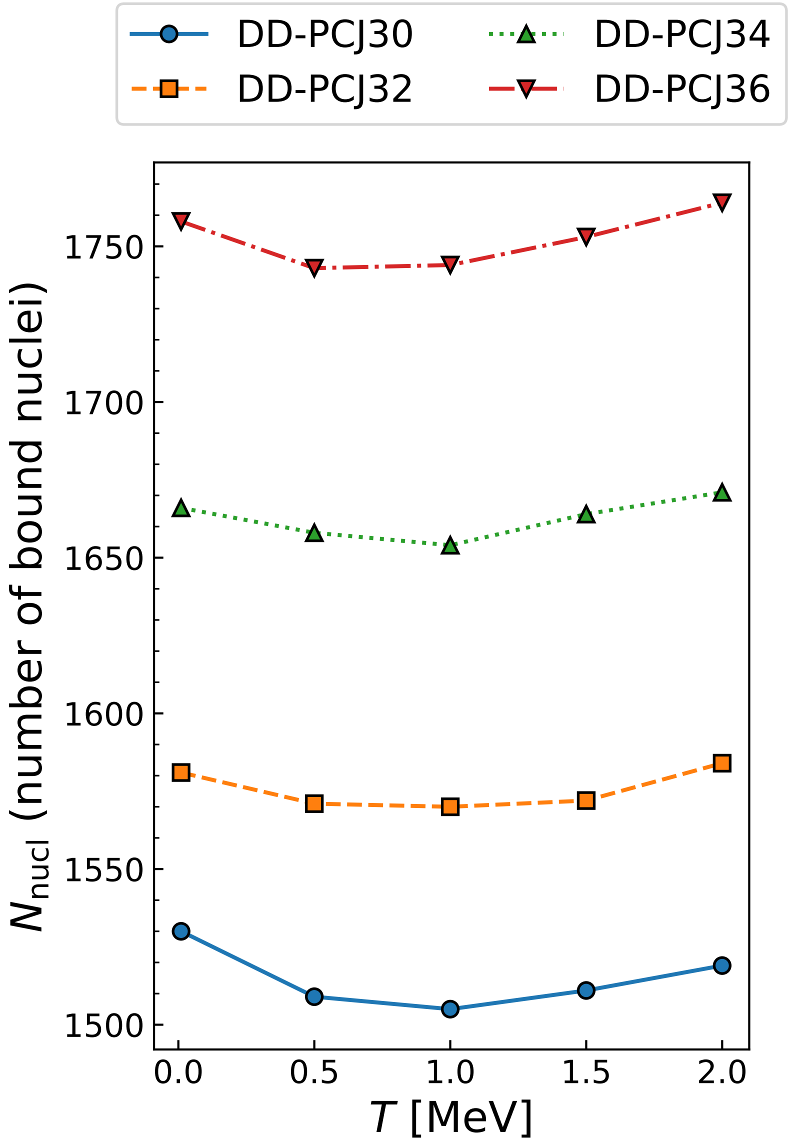


Supplementary Figure 13. Temperature dependence of the number of bound even-even nuclei $N_{\mathrm{nucl}}$, calculated with DD-PCJ30, DD-PCJ32, DD-PCJ34 and DD-PCJ36 relativistic EDFs.

## Supplementary Note 8. DRIP LINES AT FINITE TEMPERATURE

Here we list the two-proton and two-neutron drip lines calculated with three relativistic EDFs: DD-ME2, DD-PC1, and DD-PCX at $T = 0, 0.5, 0.8, 1.0, 1.2, 1.5, 1.8$, and 2.0 MeV. For a given proton number $Z$, neutron number of two-proton drip line is given by $N_{\text{min}}$ and neutron number of the two-neutron drip line is $N_{\text{max}}$. Results are shown in Supplementary Table 3–10.

Supplementary Table 3. The neutron number of the two-proton ($N_{min}$) and two-neutron ($N_{max}$) drip line for a given proton number $Z$. Results are displayed for 3 relativistic interactions: DD-ME2, DD-PC1 and DD-PCX at $T = 0$ and 0.5 MeV.

| | DD-ME2 | | | | DD-PC1 | | | | DD-PCX | | | |
|---|---|---|---|---|---|---|---|---|---|---|---|---|
| | $T = 0$ MeV | | $T = 0.5$ MeV | | $T = 0$ MeV | | $T = 0.5$ MeV | | $T = 0$ MeV | | $T = 0.5$ MeV | |
| $Z$ | $N_{\rm min}$ | $N_{\rm max}$ | $N_{\rm min}$ | $N_{\rm max}$ | $N_{\rm min}$ | $N_{\rm max}$ | $N_{\rm min}$ | $N_{\rm max}$ | $N_{\rm min}$ | $N_{\rm max}$ | $N_{\rm min}$ | $N_{\rm max}$ |
| 8 | 4 | 20 | 4 | 20 | 4 | 20 | 4 | 20 | 4 | 20 | 6 | 20 |
| 10 | 4 | 24 | 8 | 24 | 4 | 24 | 8 | 24 | 8 | 20 | 8 | 20 |
| 12 | 8 | 28 | 8 | 28 | 8 | 26 | 8 | 28 | 8 | 28 | 8 | 28 |
| 14 | 10 | 34 | 10 | 34 | 10 | 34 | 10 | 34 | 10 | 32 | 10 | 32 |
| 16 | 10 | 40 | 10 | 38 | 10 | 40 | 10 | 40 | 10 | 36 | 10 | 36 |
| 18 | 14 | 40 | 14 | 40 | 14 | 40 | 14 | 40 | 14 | 40 | 14 | 40 |
| 20 | 14 | 42 | 14 | 42 | 14 | 48 | 14 | 48 | 14 | 44 | 14 | 44 |
| 22 | 18 | 54 | 18 | 54 | 18 | 52 | 18 | 52 | 18 | 50 | 18 | 50 |
| 24 | 20 | 58 | 20 | 58 | 20 | 56 | 20 | 56 | 20 | 54 | 20 | 54 |
| 26 | 22 | 62 | 22 | 60 | 20 | 60 | 20 | 60 | 22 | 58 | 22 | 58 |
| 28 | 22 | 70 | 22 | 68 | 22 | 70 | 22 | 68 | 22 | 64 | 22 | 62 |
| 30 | 26 | 70 | 26 | 70 | 26 | 70 | 26 | 70 | 26 | 70 | 26 | 70 |
| 32 | 28 | 70 | 28 | 76 | 28 | 78 | 28 | 78 | 30 | 76 | 28 | 76 |
| 34 | 30 | 80 | 30 | 80 | 30 | 82 | 30 | 82 | 30 | 82 | 30 | 80 |
| 36 | 34 | 82 | 34 | 82 | 34 | 82 | 32 | 82 | 34 | 82 | 34 | 82 |
| 38 | 36 | 84 | 36 | 84 | 36 | 82 | 36 | 82 | 36 | 82 | 36 | 82 |
| 40 | 36 | 92 | 36 | 90 | 36 | 82 | 36 | 84 | 36 | 82 | 36 | 84 |
| 42 | 40 | 98 | 40 | 96 | 40 | 100 | 40 | 98 | 40 | 88 | 40 | 88 |
| 44 | 42 | 102 | 42 | 102 | 42 | 104 | 42 | 104 | 42 | 100 | 42 | 98 |
| 46 | 44 | 112 | 44 | 110 | 44 | 110 | 44 | 110 | 44 | 104 | 44 | 104 |
| 48 | 46 | 112 | 46 | 112 | 46 | 122 | 46 | 122 | 46 | 114 | 46 | 114 |
| 50 | 48 | 118 | 48 | 118 | 46 | 122 | 48 | 124 | 46 | 122 | 48 | 124 |
| 52 | 54 | 126 | 54 | 124 | 54 | 124 | 54 | 126 | 56 | 124 | 56 | 124 |
| 54 | 56 | 126 | 56 | 126 | 56 | 126 | 56 | 126 | 56 | 126 | 56 | 124 |

Supplementary Table 4. The continuation of Supplementary Table 3 up to $Z = 104$.

| | DD-ME2 | | | | DD-PC1 | | | | DD-PCX | | | |
|---|---|---|---|---|---|---|---|---|---|---|---|---|
| | $T = 0$ MeV | | $T = 0.5$ MeV | | $T = 0$ MeV | | $T = 0.5$ MeV | | $T = 0$ MeV | | $T = 0.5$ MeV | |
| $Z$ | $N_{\mathrm{min}}$ | $N_{\mathrm{max}}$ | $N_{\mathrm{min}}$ | $N_{\mathrm{max}}$ | $N_{\mathrm{min}}$ | $N_{\mathrm{max}}$ | $N_{\mathrm{min}}$ | $N_{\mathrm{max}}$ | $N_{\mathrm{min}}$ | $N_{\mathrm{max}}$ | $N_{\mathrm{min}}$ | $N_{\mathrm{max}}$ |
| 56 | 58 | 126 | 58 | 126 | 58 | 126 | 58 | 126 | 60 | 126 | 60 | 126 |
| 58 | 60 | 126 | 60 | 126 | 60 | 126 | 60 | 126 | 60 | 126 | 60 | 126 |
| 60 | 62 | 126 | 62 | 128 | 62 | 126 | 62 | 126 | 62 | 126 | 62 | 126 |
| 62 | 66 | 126 | 66 | 128 | 66 | 126 | 66 | 128 | 66 | 126 | 66 | 126 |
| 64 | 68 | 146 | 68 | 144 | 68 | 150 | 68 | 148 | 70 | 126 | 70 | 128 |
| 66 | 72 | 152 | 72 | 152 | 72 | 154 | 72 | 154 | 72 | 150 | 72 | 148 |
| 68 | 76 | 156 | 76 | 156 | 76 | 158 | 76 | 158 | 76 | 154 | 76 | 154 |
| 70 | 78 | 160 | 78 | 160 | 78 | 162 | 78 | 162 | 78 | 158 | 78 | 158 |
| 72 | 80 | 166 | 80 | 166 | 80 | 168 | 80 | 166 | 82 | 162 | 80 | 162 |
| 74 | 82 | 170 | 82 | 170 | 84 | 182 | 84 | 182 | 84 | 166 | 84 | 166 |
| 76 | 86 | 184 | 86 | 182 | 86 | 184 | 86 | 182 | 86 | 182 | 86 | 182 |
| 78 | 90 | 184 | 90 | 182 | 90 | 184 | 90 | 184 | 90 | 184 | 90 | 182 |
| 80 | 92 | 184 | 92 | 184 | 92 | 184 | 94 | 184 | 94 | 184 | 94 | 184 |
| 82 | 94 | 184 | 94 | 184 | 94 | 184 | 94 | 184 | 94 | 184 | 96 | 184 |
| 84 | 104 | 184 | 104 | 184 | 104 | 184 | 104 | 184 | 106 | 184 | 104 | 184 |
| 86 | 106 | 184 | 108 | 184 | 108 | 184 | 108 | 184 | 108 | 184 | 108 | 184 |
| 88 | 110 | 184 | 110 | 186 | 110 | 184 | 110 | 186 | 110 | 184 | 110 | 184 |
| 90 | 116 | 184 | 114 | 186 | 116 | 210 | 116 | 210 | 116 | 184 | 116 | 186 |
| 92 | 120 | 210 | 120 | 210 | 120 | 216 | 120 | 214 | 120 | 208 | 120 | 208 |
| 94 | 126 | 214 | 124 | 214 | 126 | 218 | 126 | 218 | 124 | 212 | 124 | 212 |
| 96 | 128 | 218 | 128 | 218 | 130 | 220 | 130 | 222 | 128 | 216 | 128 | 216 |
| 98 | 130 | 222 | 130 | 222 | 130 | 228 | 132 | 226 | 130 | 220 | 130 | 220 |
| 100 | 132 | 228 | 132 | 226 | 132 | 232 | 132 | 230 | 132 | 224 | 132 | 224 |
| 102 | 136 | 230 | 136 | 230 | 136 | 232 | 136 | 244 | 136 | 230 | 136 | 228 |
| 104 | 140 | 234 | 140 | 234 | 140 | 248 | 140 | 250 | 142 | 232 | 142 | 232 |

Supplementary Table 5. The same as in Supplementary Table 3 but at $T = 0.8$ and 1.0 MeV.

| | DD-ME2 | | | | DD-PC1 | | | | DD-PCX | | | |
|---|---|---|---|---|---|---|---|---|---|---|---|---|
| | $T = 0.8$ MeV | | $T = 1.0$ MeV | | $T = 0.8$ MeV | | $T = 1.0$ MeV | | $T = 0.8$ MeV | | $T = 1.0$ MeV | |
| $Z$ | $N_{\min}$ | $N_{\max}$ | $N_{\min}$ | $N_{\max}$ | $N_{\min}$ | $N_{\max}$ | $N_{\min}$ | $N_{\max}$ | $N_{\min}$ | $N_{\max}$ | $N_{\min}$ | $N_{\max}$ |
| 8 | 4 | 20 | 4 | 20 | 4 | 20 | 4 | 20 | 4 | 18 | 4 | 18 |
| 10 | 8 | 24 | 8 | 24 | 8 | 24 | 8 | 24 | 8 | 20 | 8 | 20 |
| 12 | 8 | 28 | 8 | 28 | 8 | 28 | 8 | 28 | 8 | 28 | 8 | 28 |
| 14 | 10 | 34 | 10 | 34 | 10 | 34 | 10 | 34 | 10 | 32 | 10 | 32 |
| 16 | 12 | 38 | 12 | 38 | 10 | 40 | 10 | 38 | 10 | 36 | 10 | 36 |
| 18 | 14 | 40 | 14 | 40 | 12 | 40 | 12 | 42 | 14 | 40 | 14 | 40 |
| 20 | 14 | 44 | 14 | 46 | 14 | 48 | 14 | 48 | 14 | 44 | 14 | 44 |
| 22 | 18 | 54 | 18 | 54 | 18 | 52 | 18 | 52 | 18 | 50 | 18 | 50 |
| 24 | 20 | 58 | 20 | 56 | 20 | 56 | 20 | 56 | 20 | 54 | 20 | 54 |
| 26 | 22 | 60 | 22 | 60 | 20 | 60 | 20 | 60 | 22 | 56 | 22 | 56 |
| 28 | 22 | 66 | 22 | 66 | 22 | 68 | 22 | 68 | 22 | 62 | 22 | 62 |
| 30 | 26 | 70 | 26 | 70 | 26 | 72 | 26 | 72 | 26 | 68 | 26 | 68 |
| 32 | 28 | 76 | 28 | 76 | 28 | 78 | 28 | 78 | 28 | 76 | 28 | 74 |
| 34 | 30 | 80 | 30 | 80 | 30 | 82 | 30 | 80 | 30 | 80 | 30 | 80 |
| 36 | 32 | 84 | 32 | 84 | 32 | 82 | 32 | 82 | 32 | 82 | 32 | 82 |
| 38 | 36 | 86 | 34 | 86 | 34 | 84 | 34 | 84 | 36 | 82 | 36 | 84 |
| 40 | 36 | 90 | 36 | 90 | 36 | 86 | 36 | 88 | 36 | 84 | 36 | 86 |
| 42 | 40 | 96 | 38 | 94 | 38 | 98 | 38 | 98 | 40 | 88 | 38 | 88 |
| 44 | 42 | 100 | 42 | 102 | 42 | 104 | 42 | 104 | 42 | 98 | 42 | 98 |
| 46 | 44 | 108 | 44 | 108 | 44 | 108 | 44 | 112 | 44 | 104 | 44 | 104 |
| 48 | 46 | 112 | 46 | 112 | 46 | 118 | 46 | 118 | 46 | 112 | 46 | 112 |
| 50 | 48 | 118 | 48 | 118 | 48 | 122 | 48 | 122 | 48 | 120 | 48 | 118 |
| 52 | 54 | 124 | 54 | 122 | 54 | 124 | 54 | 124 | 54 | 124 | 54 | 122 |
| 54 | 56 | 126 | 56 | 126 | 56 | 126 | 56 | 126 | 56 | 124 | 56 | 124 |

Supplementary Table 6. The continuation of Supplementary Table 5 up to $Z = 104$.

| | DD-ME2 | | | | DD-PC1 | | | | DD-PCX | | | |
|---|---|---|---|---|---|---|---|---|---|---|---|---|
| | $T = 0.8$ MeV | | $T = 1.0$ MeV | | $T = 0.8$ MeV | | $T = 1.0$ MeV | | $T = 0.8$ MeV | | $T = 1.0$ MeV | |
| $Z$ | $N_{\mathrm{min}}$ | $N_{\mathrm{max}}$ | $N_{\mathrm{min}}$ | $N_{\mathrm{max}}$ | $N_{\mathrm{min}}$ | $N_{\mathrm{max}}$ | $N_{\mathrm{min}}$ | $N_{\mathrm{max}}$ | $N_{\mathrm{min}}$ | $N_{\mathrm{max}}$ | $N_{\mathrm{min}}$ | $N_{\mathrm{max}}$ |
| 56 | 58 | 126 | 58 | 128 | 58 | 126 | 58 | 126 | 60 | 126 | 60 | 126 |
| 58 | 60 | 128 | 60 | 128 | 60 | 126 | 60 | 128 | 60 | 126 | 60 | 126 |
| 60 | 62 | 130 | 64 | 132 | 64 | 128 | 64 | 130 | 64 | 126 | 64 | 128 |
| 62 | 66 | 142 | 66 | 140 | 66 | 144 | 66 | 144 | 66 | 128 | 66 | 130 |
| 64 | 68 | 146 | 70 | 146 | 70 | 148 | 70 | 148 | 70 | 144 | 70 | 144 |
| 66 | 72 | 152 | 72 | 152 | 72 | 152 | 72 | 152 | 72 | 148 | 72 | 148 |
| 68 | 76 | 156 | 76 | 156 | 76 | 158 | 76 | 158 | 76 | 152 | 76 | 152 |
| 70 | 78 | 160 | 78 | 160 | 78 | 162 | 78 | 162 | 78 | 158 | 78 | 156 |
| 72 | 80 | 166 | 80 | 166 | 80 | 166 | 80 | 166 | 80 | 162 | 80 | 162 |
| 74 | 82 | 170 | 84 | 172 | 86 | 180 | 84 | 178 | 84 | 166 | 84 | 166 |
| 76 | 86 | 180 | 86 | 178 | 86 | 180 | 86 | 180 | 86 | 180 | 86 | 178 |
| 78 | 90 | 182 | 88 | 182 | 90 | 182 | 90 | 180 | 90 | 180 | 90 | 178 |
| 80 | 92 | 184 | 92 | 182 | 92 | 182 | 92 | 182 | 94 | 182 | 94 | 180 |
| 82 | 96 | 184 | 98 | 184 | 96 | 184 | 98 | 184 | 96 | 182 | 98 | 182 |
| 84 | 102 | 186 | 102 | 186 | 104 | 184 | 102 | 186 | 104 | 184 | 102 | 184 |
| 86 | 106 | 186 | 106 | 188 | 106 | 188 | 106 | 190 | 106 | 184 | 106 | 184 |
| 88 | 110 | 190 | 110 | 192 | 110 | 192 | 110 | 196 | 110 | 186 | 110 | 188 |
| 90 | 114 | 194 | 114 | 206 | 114 | 210 | 114 | 210 | 116 | 188 | 114 | 192 |
| 92 | 118 | 210 | 118 | 210 | 120 | 214 | 120 | 214 | 120 | 208 | 120 | 208 |
| 94 | 122 | 214 | 122 | 214 | 124 | 218 | 124 | 218 | 124 | 212 | 124 | 212 |
| 96 | 128 | 218 | 126 | 220 | 128 | 222 | 128 | 222 | 128 | 216 | 128 | 216 |
| 98 | 130 | 222 | 130 | 224 | 132 | 228 | 130 | 234 | 130 | 220 | 130 | 220 |
| 100 | 132 | 228 | 132 | 228 | 134 | 240 | 134 | 238 | 132 | 224 | 132 | 224 |
| 102 | 136 | 232 | 136 | 232 | 136 | 244 | 136 | 242 | 136 | 228 | 136 | 228 |
| 104 | 140 | 248 | 140 | 246 | 140 | 246 | 140 | 244 | 140 | 236 | 140 | 240 |

Supplementary Table 7. The same as in Supplementary Table 3 but at $T = 1.2$ and 1.5 MeV.

| | DD-ME2 | | | | DD-PC1 | | | | DD-PCX | | | |
|---|---|---|---|---|---|---|---|---|---|---|---|---|
| | $T = 1.2$ MeV | | $T = 1.5$ MeV | | $T = 1.2$ MeV | | $T = 1.5$ MeV | | $T = 1.2$ MeV | | $T = 1.5$ MeV | |
| $Z$ | $N_{\rm min}$ | $N_{\rm max}$ | $N_{\rm min}$ | $N_{\rm max}$ | $N_{\rm min}$ | $N_{\rm max}$ | $N_{\rm min}$ | $N_{\rm max}$ | $N_{\rm min}$ | $N_{\rm max}$ | $N_{\rm min}$ | $N_{\rm max}$ |
| 8 | 4 | 20 | 4 | 20 | 4 | 20 | 4 | 20 | 4 | 18 | 4 | 18 |
| 10 | 8 | 24 | 6 | 24 | 8 | 24 | 6 | 24 | 8 | 22 | 8 | 22 |
| 12 | 8 | 28 | 8 | 28 | 8 | 28 | 8 | 30 | 8 | 28 | 8 | 28 |
| 14 | 10 | 34 | 10 | 34 | 10 | 34 | 10 | 34 | 10 | 32 | 10 | 32 |
| 16 | 10 | 38 | 10 | 38 | 10 | 38 | 10 | 38 | 10 | 36 | 10 | 36 |
| 18 | 12 | 42 | 12 | 42 | 12 | 42 | 12 | 42 | 14 | 40 | 12 | 40 |
| 20 | 14 | 46 | 14 | 46 | 14 | 48 | 14 | 48 | 14 | 44 | 14 | 44 |
| 22 | 18 | 52 | 18 | 52 | 18 | 52 | 18 | 52 | 18 | 50 | 18 | 50 |
| 24 | 20 | 56 | 20 | 56 | 20 | 56 | 20 | 56 | 20 | 54 | 20 | 54 |
| 26 | 22 | 60 | 20 | 60 | 20 | 60 | 20 | 60 | 22 | 56 | 20 | 56 |
| 28 | 22 | 64 | 22 | 64 | 22 | 68 | 22 | 66 | 22 | 62 | 22 | 62 |
| 30 | 26 | 70 | 26 | 70 | 26 | 72 | 26 | 72 | 26 | 68 | 26 | 68 |
| 32 | 28 | 74 | 28 | 74 | 28 | 76 | 28 | 76 | 28 | 74 | 28 | 74 |
| 34 | 30 | 80 | 30 | 80 | 30 | 80 | 30 | 80 | 30 | 78 | 30 | 78 |
| 36 | 32 | 84 | 32 | 84 | 32 | 82 | 32 | 84 | 32 | 82 | 32 | 80 |
| 38 | 34 | 88 | 34 | 88 | 34 | 86 | 34 | 86 | 34 | 84 | 34 | 84 |
| 40 | 36 | 90 | 36 | 90 | 36 | 90 | 36 | 90 | 36 | 86 | 36 | 86 |
| 42 | 38 | 96 | 38 | 96 | 38 | 96 | 38 | 96 | 38 | 88 | 38 | 90 |
| 44 | 42 | 102 | 40 | 100 | 40 | 102 | 40 | 104 | 42 | 96 | 40 | 96 |
| 46 | 44 | 108 | 44 | 106 | 44 | 110 | 44 | 110 | 44 | 102 | 44 | 102 |
| 48 | 46 | 112 | 46 | 112 | 46 | 116 | 46 | 114 | 46 | 112 | 46 | 110 |
| 50 | 50 | 118 | 50 | 116 | 50 | 120 | 50 | 118 | 50 | 116 | 50 | 116 |
| 52 | 54 | 122 | 54 | 120 | 54 | 122 | 54 | 122 | 54 | 120 | 54 | 120 |
| 54 | 56 | 124 | 56 | 124 | 56 | 124 | 56 | 124 | 56 | 124 | 56 | 122 |

Supplementary Table 8. The continuation of Supplementary Table 7 up to $Z = 104$.

| | DD-ME2 | | | | DD-PC1 | | | | DD-PCX | | | |
|---|---|---|---|---|---|---|---|---|---|---|---|---|
| | $T = 1.2$ MeV | | $T = 1.5$ MeV | | $T = 1.2$ MeV | | $T = 1.5$ MeV | | $T = 1.2$ MeV | | $T = 1.5$ MeV | |
| $Z$ | $N_{\rm min}$ | $N_{\rm max}$ | $N_{\rm min}$ | $N_{\rm max}$ | $N_{\rm min}$ | $N_{\rm max}$ | $N_{\rm min}$ | $N_{\rm max}$ | $N_{\rm min}$ | $N_{\rm max}$ | $N_{\rm min}$ | $N_{\rm max}$ |
| 56 | 58 | 128 | 58 | 128 | 58 | 126 | 58 | 128 | 58 | 124 | 58 | 124 |
| 58 | 60 | 130 | 60 | 130 | 60 | 128 | 60 | 130 | 60 | 126 | 62 | 126 |
| 60 | 64 | 132 | 64 | 134 | 64 | 132 | 64 | 134 | 64 | 128 | 64 | 130 |
| 62 | 66 | 142 | 66 | 140 | 66 | 146 | 66 | 144 | 66 | 132 | 66 | 134 |
| 64 | 70 | 148 | 70 | 146 | 70 | 148 | 70 | 148 | 70 | 144 | 70 | 142 |
| 66 | 72 | 152 | 72 | 152 | 72 | 154 | 72 | 154 | 72 | 148 | 72 | 148 |
| 68 | 76 | 156 | 76 | 156 | 76 | 158 | 76 | 158 | 76 | 152 | 76 | 152 |
| 70 | 78 | 160 | 78 | 160 | 78 | 162 | 78 | 162 | 78 | 156 | 78 | 156 |
| 72 | 80 | 166 | 80 | 166 | 80 | 166 | 82 | 170 | 80 | 162 | 80 | 160 |
| 74 | 84 | 174 | 84 | 172 | 84 | 176 | 84 | 174 | 84 | 166 | 84 | 170 |
| 76 | 86 | 178 | 86 | 176 | 86 | 178 | 88 | 178 | 86 | 176 | 86 | 174 |
| 78 | 90 | 180 | 90 | 180 | 90 | 180 | 90 | 180 | 90 | 178 | 90 | 176 |
| 80 | 92 | 182 | 94 | 182 | 94 | 182 | 94 | 184 | 94 | 180 | 94 | 178 |
| 82 | 98 | 186 | 98 | 186 | 98 | 186 | 98 | 186 | 98 | 182 | 98 | 182 |
| 84 | 102 | 188 | 102 | 190 | 102 | 188 | 102 | 190 | 102 | 184 | 102 | 184 |
| 86 | 106 | 190 | 106 | 194 | 106 | 192 | 106 | 196 | 106 | 186 | 106 | 188 |
| 88 | 110 | 196 | 110 | 198 | 110 | 206 | 110 | 206 | 110 | 190 | 110 | 192 |
| 90 | 114 | 206 | 114 | 206 | 114 | 210 | 114 | 210 | 114 | 202 | 114 | 202 |
| 92 | 118 | 212 | 118 | 212 | 118 | 214 | 118 | 214 | 120 | 208 | 118 | 208 |
| 94 | 122 | 216 | 122 | 216 | 122 | 218 | 122 | 218 | 122 | 212 | 122 | 212 |
| 96 | 126 | 220 | 124 | 220 | 126 | 224 | 126 | 226 | 126 | 216 | 126 | 216 |
| 98 | 130 | 224 | 128 | 224 | 130 | 234 | 130 | 230 | 130 | 220 | 130 | 220 |
| 100 | 132 | 228 | 132 | 230 | 134 | 236 | 132 | 234 | 132 | 224 | 132 | 226 |
| 102 | 136 | 232 | 134 | 238 | 136 | 240 | 136 | 238 | 136 | 234 | 136 | 232 |
| 104 | 138 | 244 | 138 | 242 | 140 | 244 | 140 | 242 | 140 | 238 | 140 | 236 |

Supplementary Table 9. The same as in Supplementary Table 3 but at $T = 1.8$ and 2.0 MeV.

| | DD-ME2 | | | | DD-PC1 | | | | DD-PCX | | | |
|---|---|---|---|---|---|---|---|---|---|---|---|---|
| | $T = 1.8$ MeV | | $T = 2.0$ MeV | | $T = 1.8$ MeV | | $T = 2.0$ MeV | | $T = 1.8$ MeV | | $T = 2.0$ MeV | |
| $Z$ | $N_{\rm min}$ | $N_{\rm max}$ | $N_{\rm min}$ | $N_{\rm max}$ | $N_{\rm min}$ | $N_{\rm max}$ | $N_{\rm min}$ | $N_{\rm max}$ | $N_{\rm min}$ | $N_{\rm max}$ | $N_{\rm min}$ | $N_{\rm max}$ |
| 8 | 4 | 20 | 4 | 20 | 4 | 20 | 4 | 20 | 4 | 18 | 4 | 18 |
| 10 | 6 | 24 | 6 | 24 | 6 | 24 | 6 | 24 | 6 | 22 | 6 | 22 |
| 12 | 8 | 30 | 8 | 30 | 8 | 30 | 8 | 30 | 8 | 28 | 8 | 28 |
| 14 | 10 | 34 | 10 | 34 | 10 | 34 | 10 | 34 | 10 | 32 | 10 | 32 |
| 16 | 10 | 38 | 10 | 38 | 10 | 38 | 10 | 38 | 10 | 36 | 10 | 36 |
| 18 | 12 | 42 | 12 | 42 | 12 | 42 | 12 | 44 | 12 | 40 | 12 | 40 |
| 20 | 14 | 46 | 14 | 46 | 14 | 48 | 14 | 48 | 14 | 44 | 14 | 46 |
| 22 | 16 | 52 | 16 | 52 | 16 | 52 | 16 | 52 | 18 | 50 | 16 | 50 |
| 24 | 18 | 56 | 18 | 56 | 18 | 56 | 18 | 56 | 20 | 54 | 18 | 54 |
| 26 | 20 | 60 | 20 | 60 | 20 | 60 | 20 | 62 | 20 | 56 | 20 | 58 |
| 28 | 22 | 64 | 22 | 64 | 22 | 66 | 22 | 66 | 22 | 62 | 22 | 62 |
| 30 | 26 | 70 | 24 | 70 | 26 | 72 | 24 | 72 | 26 | 68 | 26 | 68 |
| 32 | 28 | 74 | 28 | 74 | 28 | 76 | 28 | 76 | 28 | 72 | 28 | 72 |
| 34 | 30 | 80 | 30 | 80 | 30 | 80 | 30 | 80 | 30 | 78 | 30 | 76 |
| 36 | 32 | 84 | 32 | 84 | 32 | 84 | 32 | 84 | 32 | 80 | 32 | 80 |
| 38 | 34 | 88 | 34 | 88 | 34 | 88 | 34 | 88 | 34 | 84 | 34 | 84 |
| 40 | 36 | 92 | 36 | 92 | 36 | 92 | 36 | 92 | 36 | 88 | 36 | 88 |
| 42 | 38 | 96 | 38 | 96 | 38 | 96 | 38 | 98 | 38 | 92 | 38 | 92 |
| 44 | 40 | 100 | 40 | 100 | 40 | 104 | 40 | 102 | 40 | 96 | 40 | 96 |
| 46 | 44 | 106 | 44 | 106 | 44 | 108 | 44 | 108 | 44 | 102 | 44 | 102 |
| 48 | 46 | 112 | 46 | 112 | 46 | 114 | 46 | 114 | 46 | 108 | 46 | 108 |
| 50 | 50 | 116 | 50 | 116 | 50 | 118 | 50 | 118 | 50 | 114 | 50 | 114 |
| 52 | 52 | 120 | 52 | 120 | 52 | 122 | 52 | 122 | 54 | 118 | 52 | 118 |
| 54 | 56 | 124 | 56 | 124 | 56 | 124 | 56 | 124 | 56 | 122 | 56 | 120 |

Supplementary Table 10. The continuation of Supplementary Table 9 up to $Z = 104$.

| | DD-ME2 | | | | DD-PC1 | | | | DD-PCX | | | |
|---|---|---|---|---|---|---|---|---|---|---|---|---|
| | $T = 1.8$ MeV | | $T = 2.0$ MeV | | $T = 1.8$ MeV | | $T = 2.0$ MeV | | $T = 1.8$ MeV | | $T = 2.0$ MeV | |
| $Z$ | $N_{\text{min}}$ | $N_{\text{max}}$ | $N_{\text{min}}$ | $N_{\text{max}}$ | $N_{\text{min}}$ | $N_{\text{max}}$ | $N_{\text{min}}$ | $N_{\text{max}}$ | $N_{\text{min}}$ | $N_{\text{max}}$ | $N_{\text{min}}$ | $N_{\text{max}}$ |
| 56 | 58 | 128 | 58 | 128 | 58 | 128 | 58 | 128 | 58 | 124 | 58 | 124 |
| 58 | 60 | 132 | 60 | 132 | 60 | 132 | 60 | 132 | 60 | 128 | 60 | 128 |
| 60 | 64 | 136 | 64 | 136 | 64 | 136 | 64 | 138 | 64 | 130 | 64 | 132 |
| 62 | 66 | 140 | 66 | 140 | 66 | 144 | 66 | 144 | 66 | 136 | 66 | 136 |
| 64 | 70 | 146 | 70 | 146 | 70 | 148 | 70 | 148 | 70 | 142 | 70 | 142 |
| 66 | 72 | 152 | 72 | 152 | 72 | 154 | 72 | 154 | 72 | 148 | 72 | 148 |
| 68 | 76 | 156 | 74 | 156 | 76 | 158 | 76 | 158 | 76 | 152 | 76 | 152 |
| 70 | 78 | 160 | 78 | 160 | 78 | 164 | 78 | 164 | 78 | 156 | 78 | 156 |
| 72 | 80 | 166 | 80 | 166 | 82 | 170 | 82 | 168 | 82 | 160 | 82 | 162 |
| 74 | 84 | 172 | 84 | 170 | 84 | 172 | 84 | 172 | 84 | 168 | 84 | 168 |
| 76 | 88 | 176 | 88 | 174 | 88 | 176 | 88 | 176 | 88 | 172 | 88 | 172 |
| 78 | 90 | 180 | 90 | 178 | 90 | 180 | 92 | 180 | 90 | 176 | 92 | 174 |
| 80 | 94 | 182 | 94 | 182 | 94 | 184 | 94 | 184 | 94 | 178 | 94 | 178 |
| 82 | 98 | 186 | 98 | 186 | 98 | 188 | 98 | 188 | 98 | 182 | 98 | 182 |
| 84 | 102 | 190 | 102 | 192 | 102 | 192 | 102 | 194 | 102 | 186 | 102 | 186 |
| 86 | 106 | 196 | 106 | 196 | 106 | 198 | 106 | 198 | 106 | 190 | 106 | 190 |
| 88 | 110 | 200 | 108 | 200 | 110 | 206 | 110 | 204 | 110 | 194 | 110 | 196 |
| 90 | 114 | 206 | 112 | 206 | 114 | 210 | 114 | 210 | 114 | 202 | 114 | 202 |
| 92 | 116 | 212 | 116 | 212 | 118 | 214 | 118 | 214 | 118 | 206 | 118 | 206 |
| 94 | 120 | 216 | 120 | 216 | 122 | 220 | 120 | 220 | 122 | 212 | 120 | 212 |
| 96 | 124 | 220 | 124 | 220 | 124 | 226 | 124 | 224 | 124 | 216 | 124 | 216 |
| 98 | 128 | 224 | 128 | 226 | 128 | 230 | 128 | 230 | 128 | 220 | 128 | 222 |
| 100 | 132 | 232 | 130 | 230 | 132 | 234 | 132 | 234 | 132 | 228 | 132 | 226 |
| 102 | 134 | 236 | 134 | 236 | 136 | 238 | 136 | 238 | 136 | 232 | 134 | 230 |
| 104 | 138 | 240 | 138 | 240 | 140 | 242 | 138 | 242 | 140 | 236 | 138 | 234 |

## SUPPLEMENTARY REFERENCES

---


[1] P. Bonche, S. Levit, D. Vautherin, Statistical properties and stability of hot nuclei, Nuclear Physics A 436 (2) (1985) 265–293. doi:https://doi.org/10.1016/0375-9474(85)90199-X. URL https://www.sciencedirect.com/science/article/pii/037594748590199X

[2] P. Bonche, S. Levit, D. Vautherin, Properties of highly excited nuclei, Nuclear Physics A 427 (2) (1984) 278–296. doi:https://doi.org/10.1016/0375-9474(84)90086-1. URL https://www.sciencedirect.com/science/article/pii/0375947484900861

[3] A. L. Goodman, Finite-temperature hfb theory, Nuclear Physics A 352 (1) (1981) 30–44. doi:https://doi.org/10.1016/0375-9474(81)90557-1. URL https://www.sciencedirect.com/science/article/pii/0375947481905571

[4] P. Ring, P. Schuck, The nuclear many-body problem, Springer Science & Business Media, 2004.

[5] P. Ring, L. Robledo, J. Egido, M. Faber, Microscopic theory of the isovector dipole resonance at high angular momenta, Nuclear Physics A 419 (2) (1984) 261–294. doi:https://doi.org/10.1016/0375-9474(84)90393-2. URL https://www.sciencedirect.com/science/article/pii/0375947484903932

[6] T. Nikšić, D. Vretenar, P. Ring, Relativistic nuclear energy density functionals: Mean-field and beyond, Progress in Particle and Nuclear Physics 66 (3) (2011) 519–548. doi:https://doi.org/10.1016/j.ppnp.2011.01.055. URL https://www.sciencedirect.com/science/article/pii/S0146641011000561

[7] P. Ring, Relativistic mean field theory in finite nuclei, Progress in Particle and Nuclear Physics 37 (1996) 193–263. doi:https://doi.org/10.1016/0146-6410(96)00054-3. URL https://www.sciencedirect.com/science/article/pii/0146641096000543

[8] Y. Tian, Z.-y. Ma, P. Ring, Axially deformed relativistic hartree bogoliubov theory with a separable pairing force, Phys. Rev. C 80 (2009) 024313. doi:10.1103/PhysRevC.80.024313. URL https://link.aps.org/doi/10.1103/PhysRevC.80.024313

[9] E. Yüksel, T. Marketin, N. Paar, Optimizing the relativistic energy density functional with nuclear ground state and collective excitation properties, Phys. Rev. C 99 (2019) 034318.

doi:10.1103/PhysRevC.99.034318.
URL https://link.aps.org/doi/10.1103/PhysRevC.99.034318

[10] E. Yüksel, T. Oishi, N. Paar, Nuclear equation of state in the relativistic point-coupling model constrained by excitations in finite nuclei, Universe 7 (3) (2021). doi:10.3390/universe7030071.
URL https://www.mdpi.com/2218-1997/7/3/71

[11] E. Yüksel, Temperature dependence of nuclear properties: A systematic study along the isotopic and isotonic chains of nuclei, Nuclear Physics A 1014 (2021) 122238. doi:https://doi.org/10.1016/j.nuclphysa.2021.122238.
URL https://www.sciencedirect.com/science/article/pii/S0375947421001032

[12] W. Pöschl, B-spline finite elements and their efficiency in solving relativistic mean field equations, Computer Physics Communications 112 (1) (1998) 42–66. doi:https://doi.org/10.1016/S0010-4655(98)00003-4.
URL https://www.sciencedirect.com/science/article/pii/S0010465598000034

[13] T. Nikšić, N. Paar, D. Vretenar, P. Ring, Dirhb—a relativistic self-consistent mean-field framework for atomic nuclei, Computer Physics Communications 185 (6) (2014) 1808–1821. doi:https://doi.org/10.1016/j.cpc.2014.02.027.
URL https://www.sciencedirect.com/science/article/pii/S0010465514000836

[14] J. Dobaczewski, W. Nazarewicz, T. R. Werner, J. F. Berger, C. R. Chinn, J. Dechargé, Mean-field description of ground-state properties of drip-line nuclei: Pairing and continuum effects, Phys. Rev. C 53 (1996) 2809–2840. doi:10.1103/PhysRevC.53.2809.
URL https://link.aps.org/doi/10.1103/PhysRevC.53.2809

[15] M. V. Stoitsov, W. Nazarewicz, S. Pittel, New discrete basis for nuclear structure studies, Phys. Rev. C 58 (1998) 2092–2098. doi:10.1103/PhysRevC.58.2092.
URL https://link.aps.org/doi/10.1103/PhysRevC.58.2092

[16] M. Stoitsov, P. Ring, D. Vretenar, G. A. Lalazissis, Solution of relativistic hartree-bogoliubov equations in configurational representation: Spherical neutron halo nuclei, Phys. Rev. C 58 (1998) 2086–2091. doi:10.1103/PhysRevC.58.2086.
URL https://link.aps.org/doi/10.1103/PhysRevC.58.2086

[17] M. V. Stoitsov, J. Dobaczewski, W. Nazarewicz, S. Pittel, D. J. Dean, Systematic study of deformed nuclei at the drip lines and beyond, Phys. Rev. C 68 (2003) 054312. doi:

10.1103/PhysRevC.68.054312.
URL https://link.aps.org/doi/10.1103/PhysRevC.68.054312

[18] D. Vretenar, A. Afanasjev, G. Lalazissis, P. Ring, Relativistic hartree–bogoliubov theory: static and dynamic aspects of exotic nuclear structure, Physics Reports 409 (3) (2005) 101–259. doi:https://doi.org/10.1016/j.physrep.2004.10.001.
URL https://www.sciencedirect.com/science/article/pii/S0370157304004545

[19] M. V. Stoitsov, J. Dobaczewski, P. Ring, S. Pittel, Quadrupole deformations of neutron-drip-line nuclei studied within the skyrme hartree-fock-bogoliubov approach, Phys. Rev. C 61 (2000) 034311. doi:10.1103/PhysRevC.61.034311.
URL https://link.aps.org/doi/10.1103/PhysRevC.61.034311

[20] Y. Zhu, J. C. Pei, Microscopic description of neutron emission rates in compound nuclei, Phys. Rev. C 90 (2014) 054316. doi:10.1103/PhysRevC.90.054316.
URL https://link.aps.org/doi/10.1103/PhysRevC.90.054316

[21] S. E. Agbemava, A. V. Afanasjev, D. Ray, P. Ring, Global performance of covariant energy density functionals: Ground state observables of even-even nuclei and the estimate of theoretical uncertainties, Phys. Rev. C 89 (2014) 054320. doi:10.1103/PhysRevC.89.054320.
URL https://link.aps.org/doi/10.1103/PhysRevC.89.054320

[22] J. Dobaczewski, W. Nazarewicz, P.-G. Reinhard, Error estimates of theoretical models: a guide, Journal of Physics G: Nuclear and Particle Physics 41 (7) (2014) 074001. doi:10.1088/0954-3899/41/7/074001.
URL https://doi.org/10.1088/0954-3899/41/7/074001

[23] Y. Gao, J. Dobaczewski, M. Kortelainen, J. Toivanen, D. Tarpanov, Propagation of uncertainties in the skyrme energy-density-functional model, Phys. Rev. C 87 (2013) 034324. doi:10.1103/PhysRevC.87.034324.
URL https://link.aps.org/doi/10.1103/PhysRevC.87.034324

[24] M. Kortelainen, J. Erler, W. Nazarewicz, N. Birge, Y. Gao, E. Olsen, Neutron-skin uncertainties of skyrme energy density functionals, Phys. Rev. C 88 (2013) 031305. doi:10.1103/PhysRevC.88.031305.
URL https://link.aps.org/doi/10.1103/PhysRevC.88.031305

[25] C. J. Horowitz, E. F. Brown, Y. Kim, W. G. Lynch, R. Michaels, A. Ono, J. Piekarewicz, M. B. Tsang, H. H. Wolter, A way forward in the study of the symmetry energy: experiment,

theory, and observation, Journal of Physics G: Nuclear and Particle Physics 41 (9) (2014) 093001. doi:10.1088/0954-3899/41/9/093001.
URL https://dx.doi.org/10.1088/0954-3899/41/9/093001

[26] W.-C. Chen, J. Piekarewicz, Searching for isovector signatures in the neutron-rich oxygen and calcium isotopes, Physics Letters B 748 (2015) 284–288. doi:https://doi.org/10.1016/j.physletb.2015.07.020.
URL https://www.sciencedirect.com/science/article/pii/S0370269315005304
